# Core-shell microcapsules compatible with routine injection enable prime-boost immunization against malaria with a single shot

**DOI:** 10.1126/scitranslmed.adw2256

**Published:** 2025-06-25

**Authors:** Romain Guyon, Sören Reinke, Adam Truby, Lee Sims, Adrian V. S. Hill, Luca Bau, Eleanor Stride, Anita Milicic

**Affiliations:** 1 The Jenner Institute, Old Road Campus Research Building, University of Oxford, Roosevelt drive, Oxford, OX3 7DQ; 2 Institute of Biomedical Engineering, Botnar Research Centre, University of Oxford, Windmill Road, Oxford, OX3 7LD

## Abstract

Inadequate booster uptake threatens the success of immunization campaigns as seen with the recently rolled-out R21 malaria vaccine. The ability to administer both prime and boost immunizations with a single injection would therefore save lives and alleviate healthcare burdens. We present a platform for delayed delivery of the booster dose that is scalable with existing technology, easily injectable, and protective against malaria in vivo. Using chip-based microfluidics, we encapsulated the R21 malaria vaccine in polymer microcapsules that release their content weeks to months post-injection. Co-injecting microcapsules with the priming dose of the R21 vaccine elicited strong antibody responses in a mouse model and provided 85% of the protection of a standard prime-boost schedule. If confirmed in humans, these results would pave the way for rapid deployment of single-shot prime-boost vaccination, an urgently needed global health intervention.

## Introduction

The success of immunization campaigns critically depends on the extent of vaccine uptake in the population. In 2022, an estimated 20.5 million children worldwide missed out on lifesaving vaccines due to missed doses (1), and global immunization coverage reached only 60% for the pneumococcal conjugate vaccine because of poor compliance (2). Achieving full protection with fewer vaccinations has been a long-standing goal in vaccinology to facilitate successful and cost-effective population protection against existing and future pathogens. In particular, the R21 vaccine against malaria, a disease responsible for 5.5% of all deaths in Africa in 2019 (3), has been recently deployed with a multiple booster schedule, and would greatly benefit from single-shot prime-boost protocols.

In the 1990s, as part of the Special Programme for Vaccine Development (4), the World Health Organization (WHO) set a goal to develop a delayed vaccine delivery system that could provide prime-boost immunization with a single injection. Over the past 30 years, numerous attempts at delayed vaccine release have used batch emulsification (BE) to encapsulate the booster dose but failed to deliver successful results (5–7). A fundamental challenge is that BE-manufactured particles, although injectable and immunogenic (8), suffer from low encapsulation efficiency, high particle heterogeneity, and antigen instability, thereby hampering clinical translation (5–7, 9, 10). Encapsulation efficiency is a key concern for cost-effective vaccine deployment (11), whereas particle heterogeneity can affect release kinetics and immunogenicity, in part due to the impact of particle size on the immune response (12, 13). Attempts to address these issues led to the development of more refined single-core core-shell systems, with improved ability to deliver delayed burst release (14, 15), higher encapsulation efficiency, sharper release profiles, and improved antigen stability. In 2017 McHugh *et al*. reported a microfabrication technology for producing poly(lactic-*co*-glycolic acid) (PLGA) cuboid particles filled with an aqueous vaccine payload (16). Although enabling precisely timed payload delivery in mice, the large (up to 400 mm), individually fabricated cubes produced by this method are incompatible with routine clinical injection, and have very limited potential for cost-effective scale-up. An alternative approach utilizes core-shell microneedles, which have been shown to confer protection in a mouse model of *Streptococcus pneumoniae* but also suffer from a non-standard inoculation protocol and limited manufacturing throughput using existing technology (17).

The key requirements of scalability and injectability have yet to be met simultaneously. Monodisperse injectable core-shell particles, potentially suitable for vaccine delivery (18), have been produced using glass capillary-based microfluidics but never successfully scaled up (19). The high throughput potential of chip-based microfluidics, on the other hand, has been repeatedly demonstrated (20, 21). However, the limitations of existing chip designs have so far precluded production of single-core microcapsules (22).

Here we describe a chip-based microfluidics method for producing vaccine-encapsulating microcapsules with a single aqueous core, of a suitable size and shape for administration by standard injection, and capable of delivering prime-boost immunization against malaria with a single injection. We encapsulated the leading clinical malaria vaccine, adjuvanted R21 (23, 24), tested its efficacy against malaria in a mouse model, and examined the effect of storage under conditions relevant to deployment in malaria-endemic countries.

## Results

### Microfluidic emulsification with a stepped chip design generates highly uniform PLGA microcapsules with high encapsulation efficiency

**Fig. 1:**
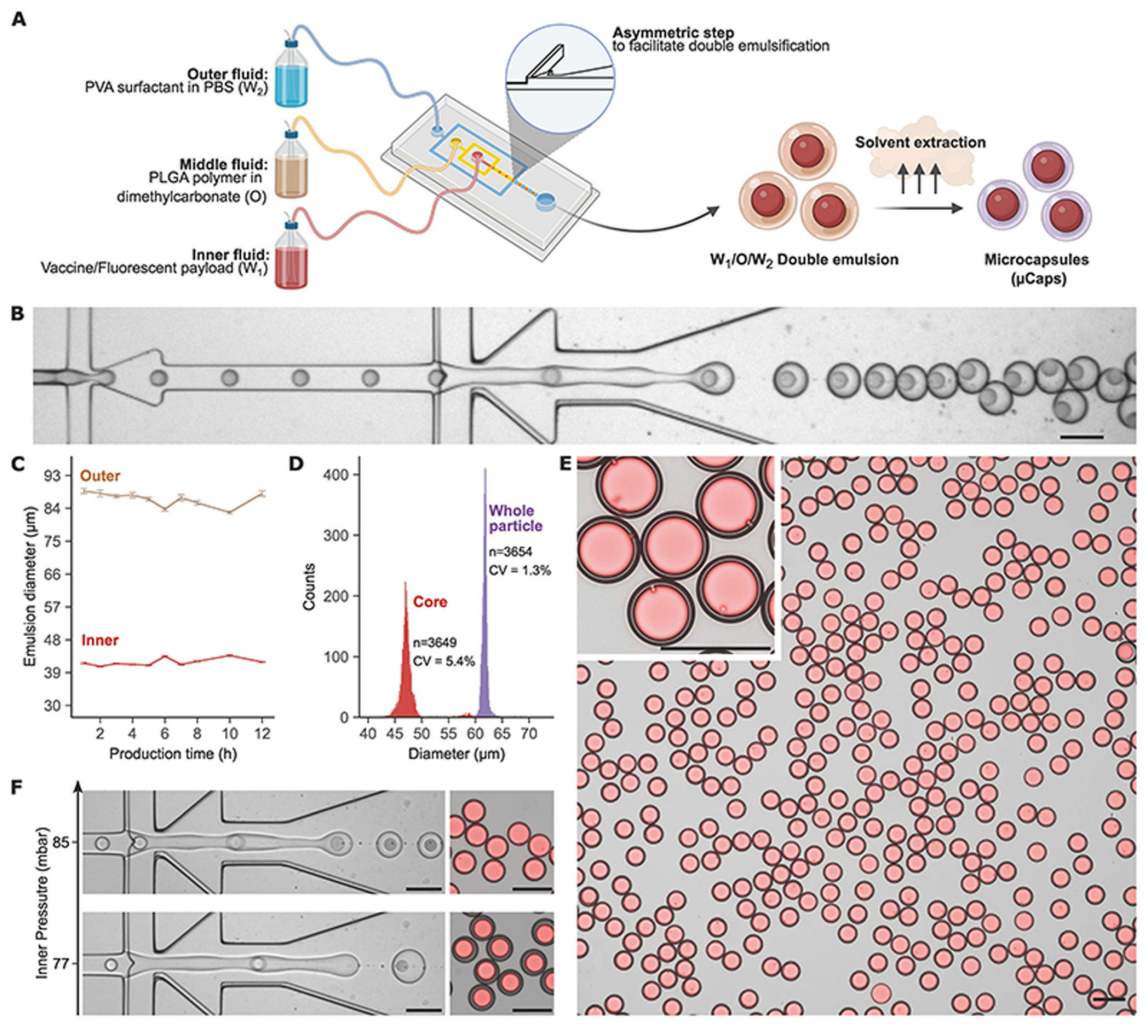
Stepped microfluidic chip design generates highly uniform PLGA microcapsules with high encapsulation efficiency. **(A)** Summary of the microfluidic W/O/W emulsification process for production of microcapsules. **(B)** High-speed camera image of the emulsification process. **(C)** Evolution of the core and outer diameters of the double emulsion during continuous production (mean ± standard deviation, n > 90 per data point). **(D)** Core and outer (whole microcapsule) diameters after solvent extraction. CV, coefficient of variation. **(E)** Fluorescence microscopy images of microcapsules produced using the stepped microfluidic design, with dextran-TRITC as a model payload and 7-17 kDa 50:50 lactide:glycolide (L:G) PLGA as the shell polymer; TRITC signal and brightfield are overlayed. Inset: magnified microcapsules. **(F)** High-speed camera images of the emulsification process at two inner fluid pressures (left) and fluorescence microscopy images of the corresponding dextran-TRITC loaded microcapsules (right); TRITC signal and brightfield are overlayed. Full range of inner fluid pressures tested is displayed in fig. S2. All scale bars are 100 µm.

We designed a double emulsion microfluidic chip for producing microcapsules that comprise an aqueous vaccine payload encapsulated within a solid PLGA shell (Fig. 1). The aqueous phase containing the payload is fed through the chip (inner fluid, Fig. 1A), emulsified by the polymer in the oil phase (middle fluid) at the first intersection, and subsequently emulsified again by the carrier aqueous phase (outer fluid) at the second intersection, to produce a water-in-oil-in-water (W/O/W) double emulsion. Addition of a step in the chip design, which provides a sudden expansion in the channel height at the second intersection (Fig. 1A and B, fig. S1 and S2, and movie S1), ensures the formation of a stable aqueous core, conferring high process stability and droplet uniformity. It reduces confinement-induced droplet deformation and prevents the newly formed double emulsion from bursting and losing the core contents (fig. S2 and movie S1).

Monitoring a continuous production run for 12 hours showed that the diameters of the core and the outer droplet remain stable (Fig. 1C), leading to highly monodisperse solidified microcapsules after solvent extraction (Fig. 1D), with >99.9% payload encapsulation efficiency (Fig. 1E). In the absence of the step, emulsification was disrupted, resulting in poor encapsulation efficiency (approximately 55% of the payload) and polydisperse solidified particles (fig. S3). By changing the pressure of the inner or the middle fluid, it was possible to modulate the core size and the shell thickness, and to create multiple-core microcapsules while preserving high uniformity and encapsulation efficiency (Fig. 1F, fig. S2). The final diameter of the solidified microcapsules, 61.8 ± 0.8 μm containing a 47.5 ± 2.5 μm aqueous core, was selected to ensure injectability using a standard vaccination needle (22-25G) (25, 26) while avoiding being phagocytosed post-vaccination (27).

### The mechanism of delayed burst release in vitro and the internal pH are dependent on the microcapsule core-shell structure and shell thickness

**Fig. 2:**
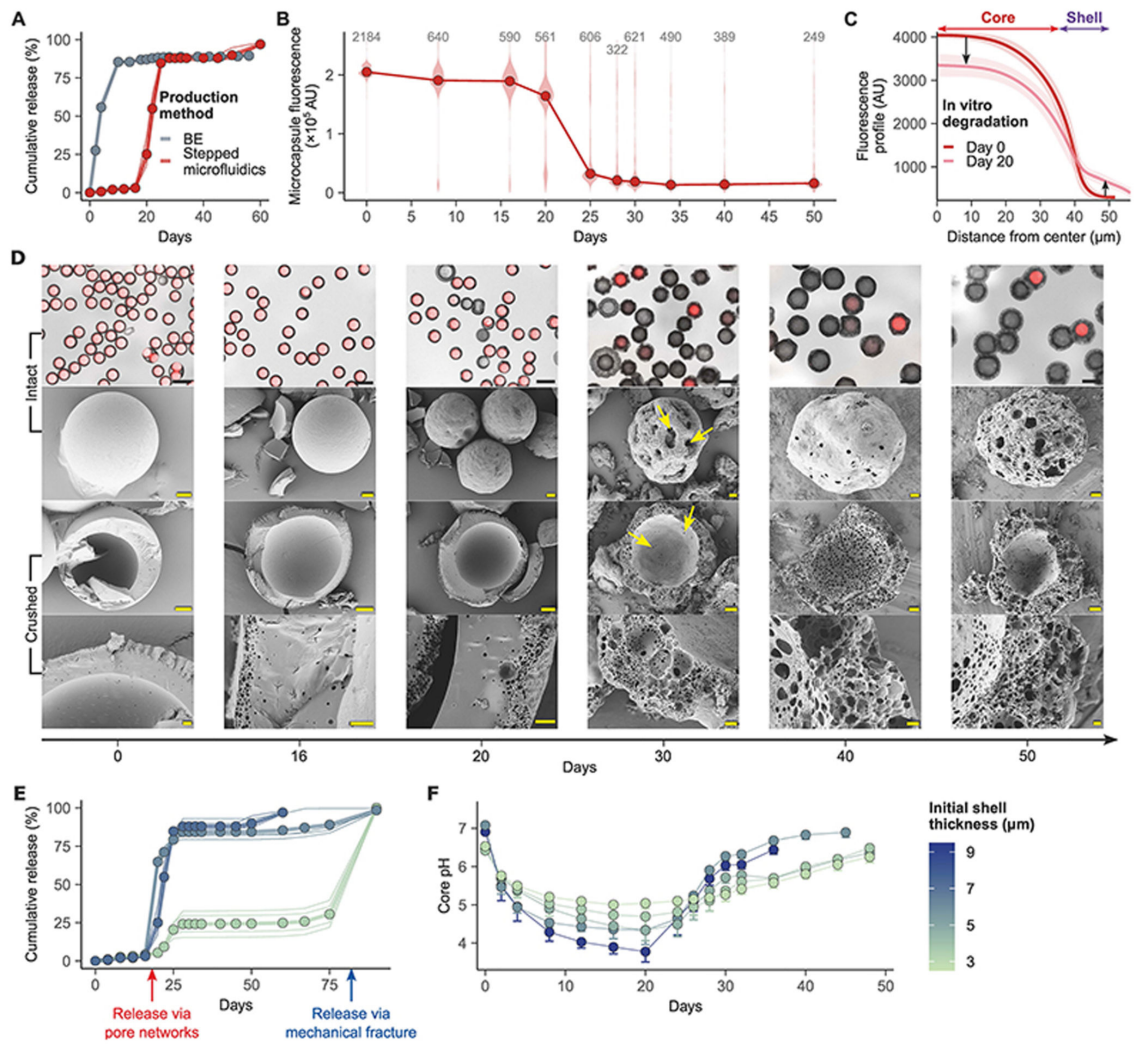
Core-shell structure of microcapsules enables delayed burst release in vitro with shell thickness-dependent release kinetics and core pH. **(A)** In vitro release kinetics at 37 °C from microcapsules manufactured by either the stepped microfluidic process (microcapsules) or batch emulsification (BE) using dextran-TRITC as a model payload and 7-17 kDa 50:50 L:G PLGA as the shell polymer. Mean of batch replicates (points joined by thick lines), and individual batch replicates (thin lines) are shown (n=4). **(B)** Evolution of microcapsule fluorescence during in vitro incubation (median and violin plot distribution, n shown above for each timepoint). AU, arbitrary units. **(C)** Cross-sectional fluorescence profile at days 0 and 20 of in vitro incubation. Mean (lines) and standard deviation (ribbons) of the profiles are shown (Day 0: n=2233, Day 20: n=454). **(D)** Fluorescence microscopy and SEM images of microcapsules containing dextran-TRITC as the model payload and PLGA 7-17 kDa 50:50 as the shell polymer at different timepoints of in vitro incubation. TRITC and brightfield are overlayed. Yellow arrows indicate holes on the shell surface. Scale bars are 100 µm (top row), 20 µm (second and third row) and 2 µm (bottom row). **(E)** Effect of varying the initial shell thickness on the in vitro release kinetics of dextran-TRITC/FITC at 37 °C. Mean of batch replicates (points joined by thick lines), and individual batch replicates (thin lines) are shown (n=4). **(F)** Evolution of core pH (mean - standard deviation, n>300 for each point) during in vitro incubation for different initial shell thicknesses.

To assess the impact of the core-shell structure and microcapsule uniformity on payload release kinetics in vitro, we compared the core-shell microcapsules with particles made by vortex BE, using fluorescently labelled dextran as model payload (Fig. 2). In contrast with the immediate release from the highly heterogeneous BE particles (fig. S3 and S4), our microcapsules displayed a clear time lag before sharply releasing most of the payload between days 20 and 22 (Fig. 2A, time to 50% release, T_50_ = 21.7 days, 95% CI 20.7-22.6). Microcapsule loading and diameter remained constant during the lag phase, followed by swelling that coincided with payload release (Fig. 2B, fig. S5A). The change in the shell/core fluorescence ratio (fig. S5B) and the cross-sectional fluorescence profile immediately before release (Fig. 2C) indicate a migration of the payload into the shell. In addition, SEM imaging reveals the formation of a pore network within the shell over time, with holes appearing on both the inner and the outer surface (Fig. 2D, yellow arrows). The suitability of dextran as a model for R21 was validated through comparison with the release kinetics of fluorescently labelled R21 (fig. S6).

Shell thickness (ranging between 3.5 and 7.2 µm) was found to have a major impact on the release kinetics (Fig. 2E). Nearly all thicker-shell microcapsules (6.1 and 7.2 µm) released the payload at approximately day 20 (T_50_ = 19 days, 95% CI 18.8-19.2 for 6.1 µm; T_50_ = 21.7 days, 95 %CI 20.7-22.6 for 7.2 µm). In contrast, most of the thinner-shell microcapsules released the payload at approximately day 80 (T_50_ = 79.1 days, 95% CI 77-81.2), likely due to mechanical fracture, as suggested by SEM imaging (fig. S7) showing comparatively fewer pores connecting to the core (fig. S8A).

The polymer shell breakdown is driven by PLGA bulk erosion through hydrolysis, resulting in the formation of acidic by-products (28). As these could affect the stability of the encapsulated antigen, we monitored the evolution of the core pH over time, using dextran-tetramethylrhodamine isothiocyanate (TRITC)/fluorescein isothiocyanate (FITC) as a pH-sensitive payload, in microcapsules with shell thickness ranging from 2.9 µm to 9.2 µm. Ratiometric fluorescence measurements (Fig. 2F) showed that the core pH dropped to a range between 4 and 5.5 around day 20, concurrently with the payload release, before increasing back towards pH 7. The minimum pH and the subsequent increase in diameter (fig. S8B) were found to correlate with shell thickness, with the smallest pH drop observed in microcapsules with the thinnest shells (Fig. 2F, fig. S8C).

### Polymer formulation controls the release kinetics in vitro and in vivo

**Fig. 3:**
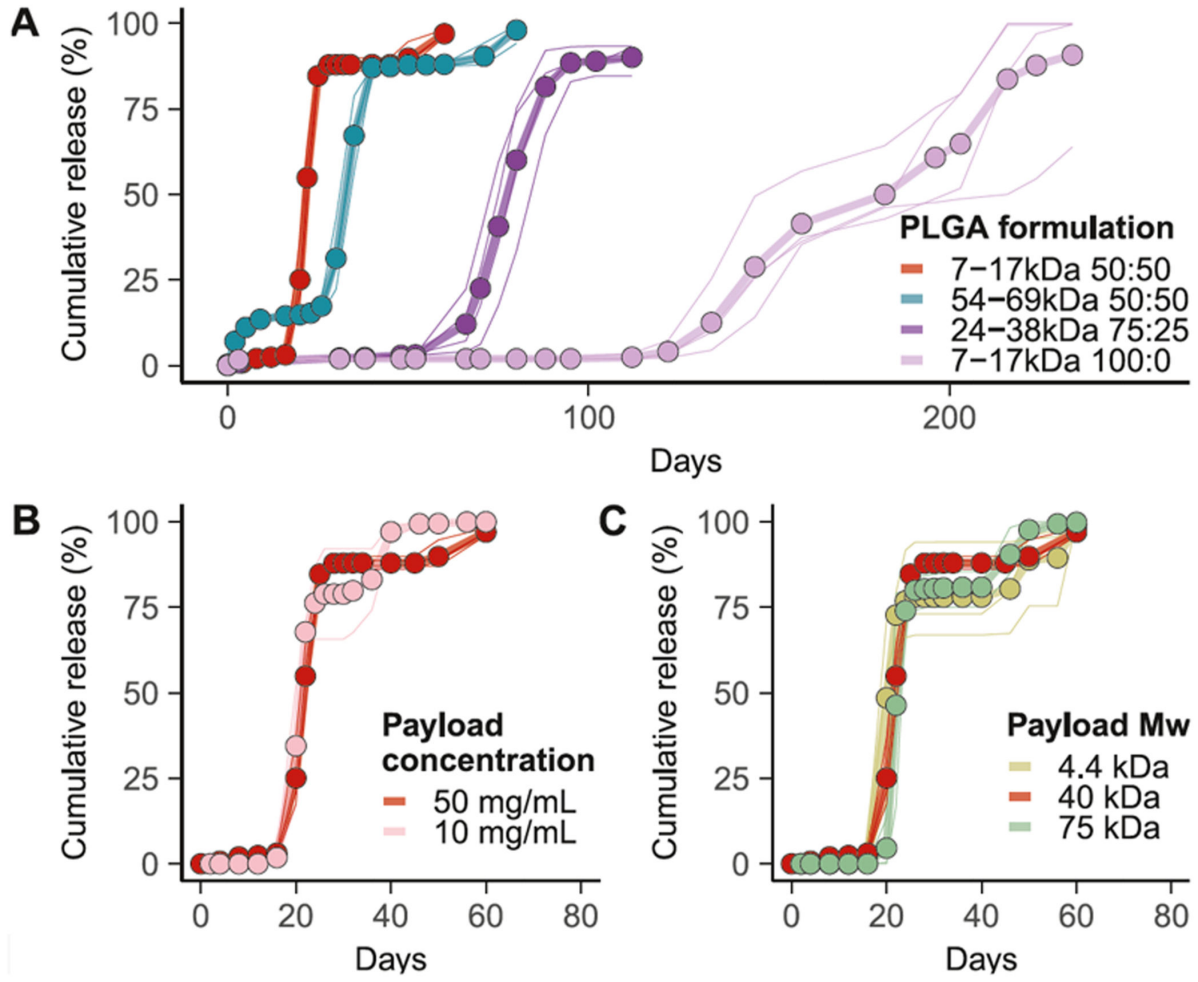
Polymer formulation controls the release kinetics in vitro. **(A)** Effect of varying MW and L:G ratio of PLGA on in vitro release kinetics using 50 mg/mL 40 kDa dextran-TRITC as the payload. **(B and C)** Release kinetics were compared with a reference formulation (7-17 kDa, 50:50 L:G, red line) after changing payload concentration (B) or payload MW (C). The release data for the reference formulation are taken from the same experiment. Mean of batch replicates (points joined by thick lines), and individual batch replicates (thin lines) are shown (n=3 or 4).

**Fig. 4:**
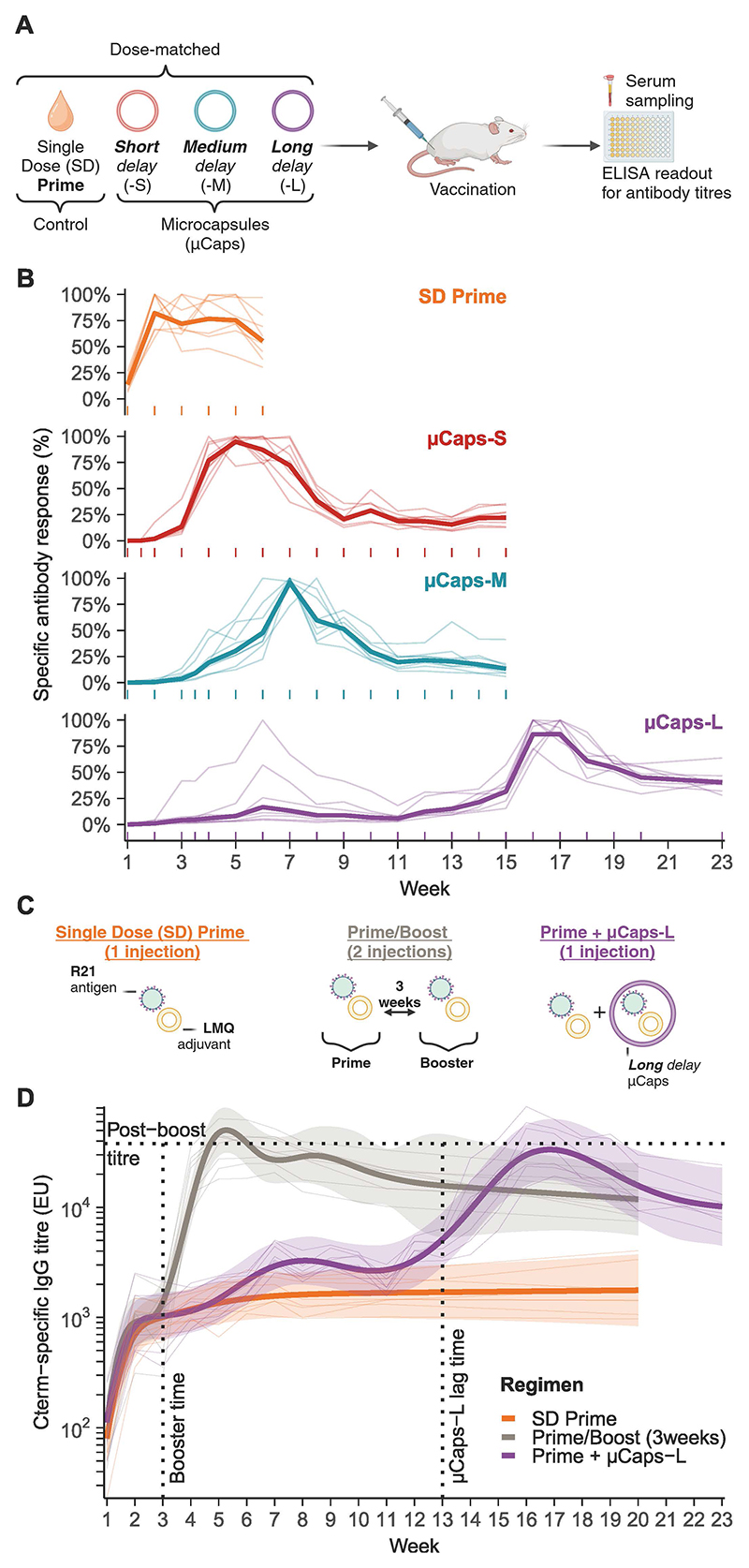
Polymer formulation determines the delayed antibody response to the encapsulated R21/LMQ malaria vaccine. **(A)** Summary of the in vivo experimental protocol. Short, medium and long delay formulations (respectively µCaps-S, µCaps-M, and µCaps-L) correspond to (7-17 kDa, 50:50 L:G), (54-69 kDa, 50:50 L:G), and (24-38 kDa, 75:25 L:G) PLGA, respectively, as presented in Fig. 3A. **(B)** Specific antibody responses for R21/LMQ vaccine encapsulated within µCaps-S, µCaps-M, or µCaps-L, compared with non-encapsulated Prime R21/LMQ vaccine control (n=8 per group). Geometric mean for each regimen (thick lines), individual responses (thin lines), and sampling timepoints (ticks) are shown. Responses are expressed as Cterm-specific antibody titers normalized to the maximum observed titer in individual mice. Absolute titers are shown in fig. S12. **(C)** Summary of the experimental protocol. * indicates a fractional (1/7th) encapsulated LMQ adjuvant dose due to the fixed concentration of the supplied adjuvant. **(D)** Cterm-specific antibody responses for different regimens of adjuvanted R21 vaccination (n=8 per group). Regimen-level responses from GAM fits (thick lines), 95% simultaneous CI (ribbons), and individual mouse responses (thin lines) are shown. Vertical dotted lines indicate the time of the booster administration in the Prime/Boost regimen, and the lag time for µCaps-L, estimated from fig. S12. The horizontal dotted line shows the IgG mean titer at week 5 (“post-boost” titer) of the Prime/Boost regimen.

Both the immunogenicity and efficacy of prime-boost vaccination critically depend on the time interval between doses (29, 30). We investigated the effect of polymer formulation and payload properties on modulating release kinetics (Fig. 3 and 4) to establish the scope of prime-boost interval control. Increasing the molecular weight or L:G ratio has been shown to delay the payload release from PLGA particles (31–33). Accordingly, we were able to extend the T_50_ of microcapsules from 22 days for low molecular weight (MW 7-17 kDa) 50:50 L:G PLGA, to 186 days for the same MW PLA (L:G = 100:0, Fig. 3A) in vitro. Lowering the concentration of dextran from 50 mg/mL to 10 mg/mL (Fig. 3B) or increasing its MW from 4.4 kDa to 75 kDa (Fig. 3C) did not affect the release kinetics. A limited amount of payload release in the first few days was deemed acceptable for further in vivo evaluation.

As physical activity can result in lactic acid accumulation in the muscle (site of injection) and lower the intramuscular pH, we investigated the effect of in vitro incubation at pH 6.8, the lowest pH reached during intense exercise (34). These conditions led to a moderate delay of payload release, increasing from 22 days at pH 7.4 to 33 days at pH 6.8 (T_50_ = 32.8, 95% CI 27.9-37.8) (fig. S9A). SEM imaging of microcapsules incubated at pH 6.8 revealed a smoother morphology and more homogeneous surface degradation (fig. S9B).

To test the in vivo controlled release performance of the microcapsules, three formulations were selected: Short (µCaps-S, red), medium (µCaps-M, blue) and long (µCaps-L, purple) delay microcapsules, which release the encapsulated payload in vitro at 22, 33 and approximately 78 days, respectively (Fig. 3A). To assess microcapsule integrity within the muscle post-injection, encapsulated fluorescently labelled dextran was used for in vivo live imaging (IVIS) (fig. S10). As non-encapsulated dextran is rapidly cleared (fig. S11), the loss of signal over time was taken as indication of payload release. The results were consistent with the order of release observed with the three formulations in vitro, with no evidence of substantial early payload release in vivo.

We then investigated the ability of these formulations to induce a delayed, booster-type immune response. We encapsulated the malaria vaccine R21 (23, 35), formulated with LMQ, an adjuvant comprising neutral liposomes, a synthetic toll-like receptor 4 (TLR4) ligand (3D6AP), and QS-21 saponin. Mice received a single intramuscular injection of a single dose (SD) of either soluble R21/LMQ (SD Prime) or encapsulated R21/LMQ (µCaps) (Fig. 4A). Longitudinal follow-up of vaccine antigen-specific antibody responses post-injection showed that the generation of IgG titers against the R21 C-terminus (Cterm) epitope was delayed for all three formulations (Fig. 4B), reaching 50% of SD Prime peak titers (fig. S12) at different times (lag time), in line with the order of release observed in vitro (Fig. 3A). SD Prime, µCaps-S, µCaps-M and µCaps-L exhibited lag times of 1.8 weeks (95% CI 1.6-2), 2.2 weeks (95% CI 2.1-2.4), 3.4 weeks (95% CI 2.7-4.4) and 13 weeks (95% CI 5.3-15.7), respectively.

To better assess the delay in the immune response, prime dose co-injected with long delay microcapsules (Prime+µCaps-L) was compared to a single prime dose (SD Prime) and a 3-week Prime/Boost regimen which provided the “pre-release” and “post-boost” titer reference values (Fig. 4C). The Prime+µCaps-L response remained close to SD Prime for about 3 months before starting to rise towards the post-boost titer (Fig. 4D). This is consistent with the lag time observed previously with µCaps-L (Fig. 4B, fig. S12).

### Single co-injection of soluble prime and encapsulated booster dose promotes immune response equivalent to the standard prime/boost regimen

**Fig. 5:**
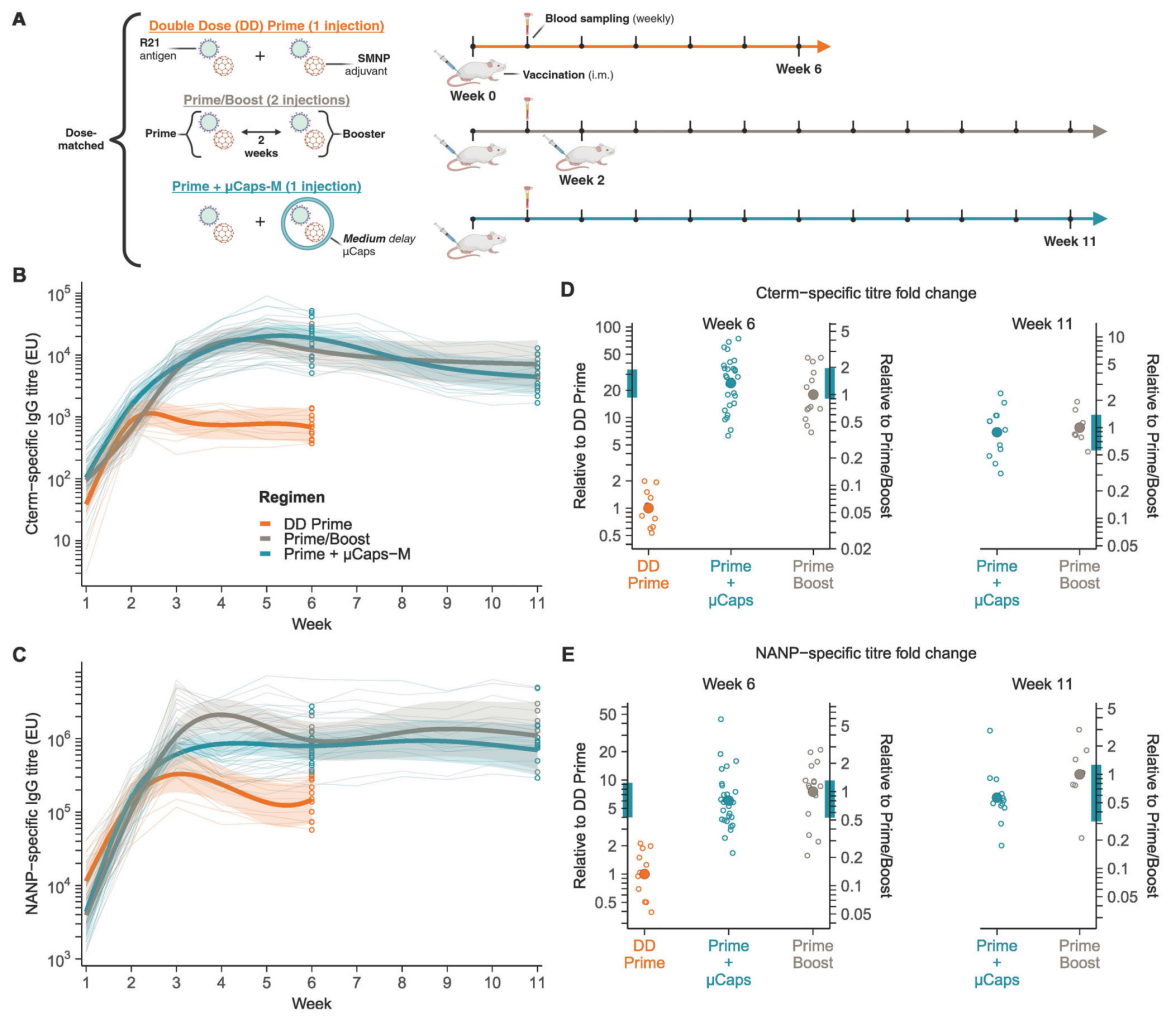
A single injection of Prime+µCaps-M induces similar immunogenicity to the standard Prime/Boost regimen. **(A)** Summary of the experimental protocol. **(B and C)** Cterm-specific (B) and NANP-specific (C) antibody titers for different regimens of dose-matched adjuvanted R21 vaccination (Week 1-6: DD Prime n=11, Prime+µCaps-M n=28, Prime/Boost n=15; Week 7-11: Prime+µCaps-M n=12, Prime/Boost n=7). The study was stopped for each mouse at the corresponding time of challenge (week 6 or week 11, Fig. 6). Regimen-level responses from GAM fits (thick lines), 95% simultaneous CI (ribbons), individual responses (thin lines), and antibody titers at time of challenge (circles) are shown. **(D and E)** Cterm-specific (D) and NANP-specific (E) titer fold changes of Prime+µCaps-M compared with geometric mean titers of DD Prime or Prime/Boost at Weeks 6 and 11 are plotted with the individual mouse data (open circles) and their geometric means (closed circles). Bootstrap 95% CIs are displayed as colored bands on the fold change axis.

To assess the immune-boosting effect of the encapsulated vaccine, mice were immunized with a single injection of soluble adjuvanted R21 combined with an encapsulated homologous booster dose in Medium delay microcapsules (Prime+µCaps-M), the formulation that most closely matches the interval between doses in the primary R21 human immunization schedule (36). This regimen was compared to a double dose prime (DD Prime) and a standard Prime/Boost regimen (two injections, two weeks apart), all dose-matched with the Prime+µCaps-M regimen. The prime-boost interval of two weeks was chosen to closely match the interval between the lag time of the SD Prime and the lag time of mCaps-M (Fig. 4B). Independent studies were conducted, using either a saponin/MPLA nanoparticle adjuvant (SMNP, Fig. 5A) or the LMQ adjuvant (fig. S13A), and monitoring the immune response over 6 to 11 weeks.

Vaccine-specific IgG titers against both the Cterm and the NANP epitope of R21 reached similar values across all regimens during the first two weeks post-injection. Subsequently, the titers in the Prime/Boost and Prime+µCaps-M groups continued to rise, whereas the titers in the DD Prime group plateaued (Fig. 5, B and C). By week 6 post injection, the Prime+µCaps-M regimen had induced non-inferior antibody titers in comparison to the standard Prime/Boost regimen for both epitopes, with a 1.3-fold change in Cterm-specific antibody titers (n_Prime+µCaps-M_ = 28, n_Prime/Boost_ = 15, 95% CI 0.9-2, p = 0.0013) and a 0.8-fold change in NANP-specific antibody titers (n_Prime+µCaps-M_ = 28, n_Prime/Boost_ = 15, 95% CI 0.5-1.3, p = 0.02) over standard Prime/Boost (Fig. 5, D and E). Mice receiving Prime+µCaps-M had a significantly higher immune response than mice immunized with DD Prime, with Cterm-specific antibody titers 24-fold higher (n_Prime+µCaps-M_ = 28, n_DD Prime_ = 11, 95% CI 16.7-38.9, p = 5□10^-6^) and NANP-specific antibody titers 6-fold higher (n_Prime+µCaps-M_ = 28, n_DD Prime_ = 11, 95% CI 4-9.3 p = 5□10^-6^). Moreover, the encapsulated booster dose induced sustained antibody titers against Cterm and NANP for up to 11 weeks, with titers throughout comparable to the standard Prime/Boost regimen (Fig. 5, D and E). Similar results were observed with LMQ as the adjuvant (fig. S13). Empty microcapsules co-injected with R21 did not have an adjuvant effect (fig. S14).

### The encapsulated booster dose offers high protective efficacy with a single injection

**Fig. 6:**
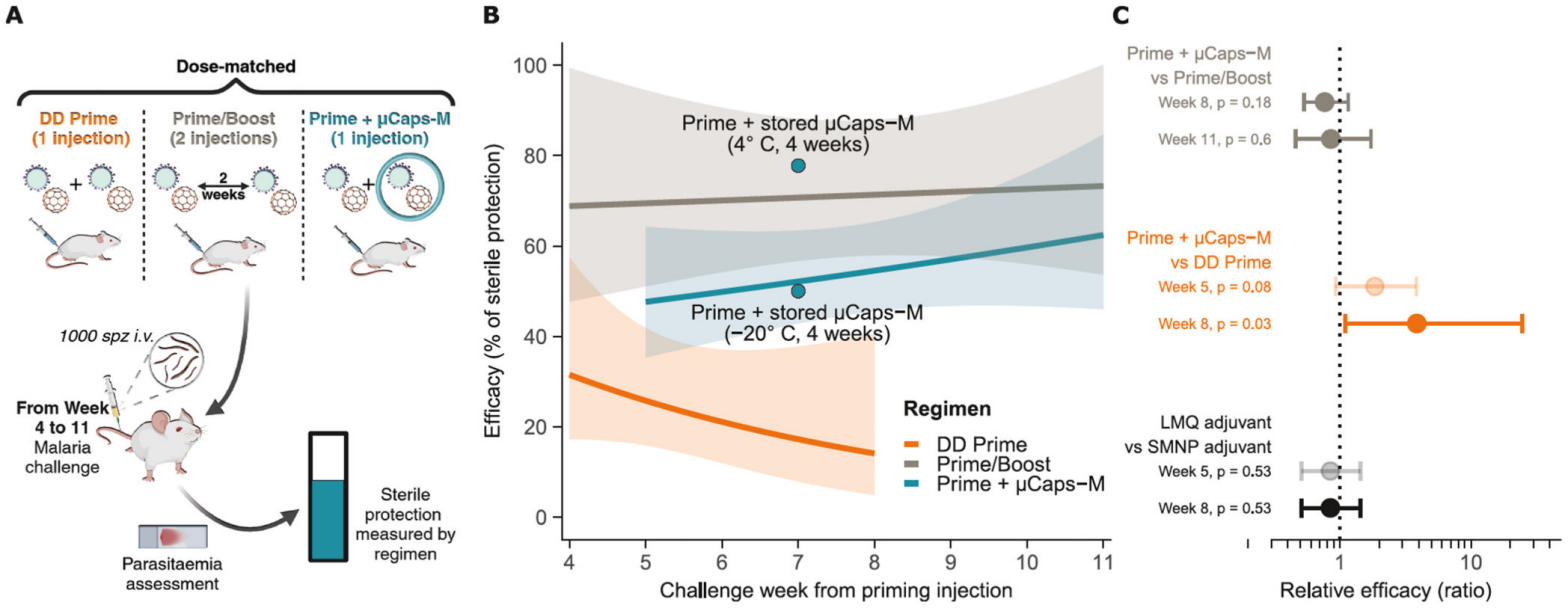
The microcapsule-delivered booster dose elicits protective efficacy against malaria in mice. **(A)** Summary of the experimental protocol. Spz, sporozoites; i.v., intravenous. **(B)** Predicted sterile efficacy against malaria over time post priming injection, calculated from cumulative data using a log-binomial regression model with adjuvant as a covariate. Predicted efficacy for each regimen, with analysis conditional on SMNP adjuvant (thick lines), and the corresponding 83.4% confidence bands (ribbons) are shown. Sterile protection rates observed after microcapsule storage at 4 °C and -20 °C are shown as dots, for reference. Corresponding raw data are displayed in fig. S15. **(C)** Relative contribution of different parameters to the protective efficacy at weeks 5 and 8 from the priming injection (log-binomial regression). Estimated ratios (dots) relative to the SMNP reference adjuvant are shown, along with error bars indicating the profile likelihood 95% CIs, and p-values (p) computed from the likelihood ratio test.

The efficacy of the Prime+µCaps-M regimen was tested in a mouse model of lethal malaria challenge by intravenous injection of transgenic *Plasmodium berghei (Pb)* sporozoites carrying the *P. falciparum* circumsporozoite (CSP) gene. Mice received DD Prime, Prime+µCaps-M, or Prime/Boost regimens of adjuvanted R21 and were challenged with malaria between week 4 and 11 to assess the durability of protection with different regimens, with subsequent assessment of parasitemia in the blood (Fig. 6A). Results from all experiments (fig. S15) were analyzed by log-binomial regression to estimate the effect of the timing of challenge on the protective efficacy for each regimen (Fig. 6B).

At 8 weeks from the priming injection, the efficacy of the Prime+µCaps-M regimen was significantly higher than the equivalent prime only dose by 3.9-fold (95% CI 1.1-24.6, p = 0.03), and reached 76% (95% CI 53-116) of the efficacy of the Prime/Boost regimen. After 11 weeks post priming injection, the efficacy of the Prime+µCaps-M regimen rose to 85% (95% CI 46-173) of the standard Prime/Boost (Fig. 6C). Importantly, the efficacy of Prime+µCaps-M showed an upward trend over time, increasing from 48% at week 5 to 62% at week 11, significantly above that of DD Prime, which offered only 14% efficacy at week 8 (p = 0.03, Fig. 6B), whereas the Prime/Boost regimen plateaued at around 70% efficacy (Week 4: 69%, Week 11: 73%).

### Microcapsules meet technical requirements for deployment of R21 vaccine in malaria-endemic countries

Logistical immunization challenges and resource scarcity in the field require vaccine formulation to be compatible with standard storage (cold-chain or dry powder) and injectability requirements. Neither storage at 4 °C for 60 days nor shelf-drying post-production (without storage), affected the in vivo release kinetics of a dextran-TRITC payload compared to freshly prepared microcapsules (storage: T_50_ = 21.4 days, 95% CI 20.9-21.8; shelf-dry: 23.8 days, 95% CI 22.7-24.8; fig. S16, A and B). Shelf-drying resulted in osmotic shrinkage of the aqueous core (fig. S16C) which reverted fully upon resuspension of microcapsules in buffer (PBS + 0.01% Tween-80, 37°C). The effect of refrigeration on the encapsulated vaccine was assessed by testing the immunogenicity of microcapsules containing R21/SMNP stored at 4 °C for 4 weeks and mechanically crushed before injection. Both NANP-specific and Cterm-specific antibody responses were comparable to a dose-matched non-encapsulated single dose prime (SD Prime) regimen (fig. S16, D and E), demonstrating that immunogenicity was not affected by cold storage.

Preservation of the delayed booster release post-storage was assessed in vivo by co-injecting stored microcapsules with a freshly prepared soluble priming vaccine (Prime+stored µCaps-M). Microcapsules stored at 4 °C for 4 weeks induced antibody titers comparable to the Prime/Boost regimen (fig. S16F), and storage at -20 °C led to a slight reduction in immunogenicity, although antibody titers were still higher than the DD Prime regimen (fig. S16G). Most importantly, vaccine efficacy was not affected by storage at 4 °C, offering up to 78% sterile protection against malaria at 7 weeks post priming injection (Fig. 6B).

Injectability, another key requirement for vaccine delivery (25, 26), was assessed by measuring the recovery of encapsulated dextran-TRITC after injection. The microcapsules were injectable through 27G and 25G needles, delivering respectively 86% (95% CI 66-105%) and 94% (95% CI 93-96%) of the target dose (fig. S16H).

## Discussion

This study presents a scalable microfluidic system for producing encapsulated vaccine core-shell formulations capable of programmable release of the R21 malaria vaccine up to several weeks post-injection. The process was optimized to produce monodisperse core-shell microcapsules approximately 65 µm in diameter to ensure injectability with 25 or 27G needles (260 and 210 µm internal diameter, respectively) while avoiding phagocytosis in vivo (37). A critical element in the development of this technology was the addition of a step in the height of the microfluidic channel to minimize wall-drag effects, resulting in greater than 99.9% encapsulation efficiency. Absence of the step led to the bursting and splitting of the double emulsion due to the drag exerted by the channel walls, as previously reported (38–40), causing reduced encapsulation efficiency and broader capsule size distribution.

The PLGA shell degrades by autocatalytic heterogenous bulk erosion, creating a network of pores which over time become increasingly interconnected. Eventually, a continuous path wide enough to allow rapid diffusion of the payload forms between the microcapsule core and the external environment (41). The stochastic nature of this process is demonstrated by the observation that almost all microcapsules are either fully loaded or fully empty at any timepoint (Fig. 2B, fig. S7A), indicating that fast, complete, release occurs from individual microcapsules at different times. The probability distribution of these individual release times, which depends on the PLGA formulation, determines the release kinetics observed in vitro. The fraction of payload released from a population of microcapsules corresponds to the fraction of microcapsules that have released, and, in turn, to the cumulative distribution function of individual release times.

Shell thickness was found to be an important parameter in controlling payload release, with thinner-shell formulations releasing in two stages (Fig. 2E). These seemingly biphasic release kinetics, however, are not a reflection of the behavior of individual microcapsules. We hypothesize that they are in fact linked to the removal of acidic by-products of PLGA degradation, as evidenced by increased core acidity and formation of larger pores in thicker-shell microcapsules. A fast release route becomes available for the acidic by-products before it does for the much larger payload, and the autocatalytic activity is substantially reduced for both thick and thin shell microcapsules, drastically decelerating pore growth and therefore the probability of pore interconnection. By the time the larger pores in thicker shells have formed a continuous path for payload release in nearly all microcapsules, only a small fraction of thinner-shell microcapsules has released due to a lower probability of interconnection for smaller pores. Similar effects were observed in monolithic PLGA particles, where both the pore size (42, 43) and acidity (44) were found to correlate with the distance from the surface. Further reinforcing this conclusion, the core pH rises at a thickness-dependent rate, with thicker-shell microcapsules re-equilibrating faster with the external environment. The effect of shell thickness on limiting acidic build-up in the core is a key feature which could be harnessed to encapsulate pH-sensitive vaccines.

The delayed immune response to adjuvanted R21 vaccine encapsulated into different microcapsule formulations reflects the delayed release observed in vitro with the same microcapsules, providing proof-of-concept for controlling the prime-boost interval with this technology. Stronger antibody responses against the Cterm epitope were observed with encapsulated vaccine and could be the result of a more sustained antigen exposure (45, 46). Due to the stochastic nature of the release process, the release phase can span days to weeks depending on the polymer formulation. Thus, at the scheduled booster interval, different microcapsule fractions would provide phased exposure to the vaccine, producing overall higher titers of antigen-specific antibodies compared to an injected booster (47). The peak titer of µCaps-L, although still comparable to the prime, was lower than the peak titer of the other microcapsule formulations. This could be due to µCaps-S and µCaps-M, but not µCaps-L, releasing the vaccine within the stability window of one month at 37°C that was measured for AS01, an LMQ-like liposomal adjuvant (48).

When co-injecting a priming vaccine with microcapsules containing the booster dose, the delayed booster release provided a substantial increase in antibody titers, comparable to a dose-matched 2-week prime/boost regimen. Antibody titers against the Cterm epitope have been shown to increase with each subsequent antigen exposure (45) and are therefore good indicators of a booster dose delivery. In contrast, NANP titers only display a modest increase after repeated dosing likely due to a self-limiting feedback in antibody generation (45).

The efficacy of the single-shot prime-boost regimen was high and durable, up to approximately 4 times that of a dose-matched prime only, and 85% of the two-shot prime-boost control, with no sign of diminishing protection for at least 11 weeks. When balanced against low patient compliance, even a relative efficacy of approximately 80% compared to a regimen requiring multiple injections can substantially improve the effectiveness of a vaccination program, especially when more than one booster dose is required. For example, uptake of the four required doses of the RTS,S malaria vaccine, which is similar to R21, was as low as 36 to 52% in Ghana, Kenya, and Malawi (49). Moreover, efficacy was retained under storage conditions relevant to vaccine deployment in countries endemic for malaria, with 78% protection achieved with microcapsules stored at 4 °C for 4 weeks. One reason for this stability could be that the R21 vaccine itself has a shelf life of 24 months at 2-8 °C in liquid form (50).

High encapsulation efficiency, high process stability during continuous production, and a chip-based approach represent further milestones towards scaling up manufacturing. In contrast with current state-of-the-art platforms for delayed vaccine delivery (16, 17), the process presented here could be implemented at a very large scale with minor modifications to existing technology (20, 51). Multiple-booster formulations could be prepared at, or close to, point-of-care by simply combining different lag time microcapsules to achieve tailored vaccination schedules. Although more thermolabile and pH-sensitive vaccines, such as mRNA-LNP formulations, might not be compatible with this technology, it is plausible that the microcapsules could be used to deliver other VLP-type vaccines, especially if based on the hepatitis B virus surface antigen, and potentially other soluble protein-in-adjuvant, viral vector-based, and inactivated vaccines.

Some limitations of this study should be noted. Although the µCaps-M based formulations did not exhibit any obvious loss of immunogenicity in vivo, characterizing the stability of the encapsulated vaccine after long-term in vitro incubation of the microcapsules could provide useful insight for the optimization of formulations with longer boosting delays. This would facilitate screening of stabilizing excipients, if required, such as the basic salts that have been used in BE-based systems (52). Another limitation is the use of fluorescent dextran for in vivo imaging, which might not accurately reflect the release kinetics of adjuvanted R21. To gain further mechanistic insight, future work should employ a labelled vaccine and fluorescent adjuvant to link the release kinetics of both components with immunogenicity and efficacy. Finally, our immunology work is limited to the monitoring of the antibody response to microcapsule-based regimens. A more extensive study of germinal center B cell activity would be important in the future to fully characterize the effect of the microcapsules on the magnitude and duration of the humoral response. Altogether, these results make a compelling argument for the feasibility of testing single-shot prime-boost vaccination clinically in a short time frame, offering a much-needed intervention in support of malaria vaccination programs.

## Materials and methods

### Study design

The objective of this study was to evaluate the in vitro release kinetics from delayed delivery microcapsules and the in vivo immunogenicity and efficacy of microcapsules encapsulating the R21 vaccine in a BALB/c mouse model of malaria. Mice were maintained at the Wellcome Centre for Human Genetics or the John Ratcliffe Hospital, University of Oxford, housed under Specific Pathogen Free (SPF) conditions and in accordance with the recommendations of the UK Animals (Scientific Procedures) Act 1986 and ARRIVE guidelines. Protocols were approved by the University of Oxford Animal Care and Ethical Review Committee for use under Project Licenses P9804B4F1 or PP0984913B granted by the UK Home Office.

The sample size for antibody titers noninferiority tests was chosen based on a power analysis for a minimum fold change of interest of 1. Assuming log antibody titers to be normally distributed with a standard deviation of 0.5 log antibody titers (obtained from pilot NANP immunogenicity data), and n=7 mice in the Prime/Boost group, we calculated that n=16 mice were needed in the Prime+µCaps-M group to achieve 80% power. Reducing the number of mice to 12 resulted in only a small loss of power (75%), which was deemed acceptable for optimal resource allocation. For all other in vivo experiments, the sample size was chosen based on published preclinical studies in the same malaria mouse model (53). All in vivo experiments were not blinded, and vaccine regimen allocation was randomized across mice. Data points represent biological replicates for in vivo experiments and microcapsule batch replicates for in vitro experiments. All relevant data are shown, and no data were excluded from the analysis.

### R21 adjuvanted vaccine

The virus-like particle R21 vaccine was produced as described previously (35). The LMQ adjuvant was by kindly provided by the Vaccine Formulation Institute (VFI), manufactured as described previously (54), and contains QS-21 saponin and the synthetic TLR4 ligand 3D-6-acyl-PHAD incorporated into neutral liposomes composed of 1,2-dioleoyl-*sn*-glycero-3-phosphocholine (DOPC) and cholesterol. The R21/LMQ mouse priming dose was defined as 1 µg of R21, 5 µg of QS21 and 2 µg of 3D6AP, and the booster dose (encapsulated in microcapsules or given as a booster injection) was defined as 1 µg of R21, 0.7 µg of QS21 and 0.3 µg of 3D6AP. The saponin/MPLA nanoparticles (SMNP) adjuvant was generously supplied by D. Irvine (MIT) and contains the TLR4 agonist Monophosphoryl lipid A (MPLA) incorporated into self-assembled complexes of saponin, cholesterol and phospholipids. Both priming and booster dose of R21/SMNP were defined as 0.5 µg of R21 and 0.5 µg of MPLA. For all experiments, double dose prime (DD Prime) regimens combined the respective priming and booster doses in a single injection. When unadjuvanted (fig. S14), the dose of R21 was defined as 1µg.

### Fluorescent labelling of the R21 vaccine

R21 was labelled with Alexa Fluor 647 (AF-R21) to enable the measurement of encapsulation efficiency and microcapsule core size post encapsulation by microfluidics. Buffer exchange of a 0.5 mL R21 stock solution (0.38 mg/mL in Tris Buffer) was performed using an ultra-centrifugal filter unit (30 kDa cut-off, Amicon, Sigma-Aldrich, #UFC503008) and centrifuged 3 times at 10,000 g for 10 minutes at 4 °C using phosphate buffer saline (PBS) as the replacing buffer. R21 in PBS was then mixed in a 0.5 mL Eppendorf tube with 50 μL of 4 mg/mL Alexa Fluor 647 NHS ester (Thermo Fisher, #A20006) suspended in dimethyl sulfoxide and vortexed for 30 seconds at 3,000 rpm. The labelling reaction was incubated on a rotating shaker for 1.5 hours at 30 rpm mix mode, 28 °C, protected from light. Removal of unincorporated dye and washing of the labelled product was done by 6 cycles of diafiltration (30 kDa cut-off, Amicon, Sigma-Aldrich, #UFC503008) at 10,000 g for 10 minutes at 4 °C using PBS. Labelled R21 was stored at -80 °C.

### R21 loaded microcapsule formulation and quality control

Quality control of the produced microcapsules was performed by microscopic image quality control, as described in the supplemental materials and methods, using the AF-R21 as the fluorescent marker within the core. Quantification of the encapsulated R21 was performed by mechanically breaking the microcapsules shells. 500 μL of the produced microcapsules batch was added to 500μL of PBS and homogenized for 30 seconds with a cell homogenizer (IKA T10 Basic Ultra Turrax Homogenizer, Cole-Parmer) to break up the microcapsules. Released R21 was isolated from the PLGA microcapsules debris by centrifugation at 15,000 g for 10 minutes, and the concentration of R21 in the supernatant was determined using a micro–BCA Protein assay (Thermo Fisher, #23235). From this, the number of microcapsules required to achieve a vaccine mouse dose was calculated (in the order of 100,000 microcapsules per mouse). Depending on the regimen, the correct number of microcapsules, antigen in solution and adjuvant were resuspended in 0.25% (w/v) medium viscosity carboxymethylcellulose (CMC, Sigma-Aldrich). CMC was added to prevent early sedimentation of microcapsules.

### Mouse immunizations

Six to 10-week-old female inbred BALB/c mice (#162, Envigo, UK) were immunized intramuscularly with a total volume of 50 μL in the tibialis muscle under 3% isoflurane anesthesia. All injections were performed with 100 μL Gas Tight glass syringes (Hamilton), with 1.40 mm barrel inner diameter to improve the microcapsule injectability (55). Serum was obtained by collecting blood from the lateral tail vein and stored at −20 °C until use.

### Live imaging of in vivo microcapsule localization (IVIS imaging)

Following intramuscular immunization of BALB/c mice with approximately 50,000 microcapsules loaded with 50 mg/mL dextran-TRITC, mice were imaged at different timepoints to follow the evolution of the fluorescence at the site of injection using the IVIS imaging system (IVIS Spectrum in vivo imaging system, PerkinElmer). Mouse lower body fur, which emits autofluorescence under IVIS imaging, was removed before each imaging session by applying hair removal cream (Veet). Images were taken under 3% isoflurane anesthesia, using excitation and emission filters of 520-550 nm and 570-590 nm, respectively.

### Enzyme-linked immunosorbent assay (ELISA) for measuring antibody titers

NANP or C-terminal (Cterm)-specific IgG ELISAs to detect antibodies against the central repeat (NANP) or the Cterm regions of CSP were performed as previously described (35). Nunc-Immuno Maxisorp 96 well plates were coated with 2μg/mL NANP6 peptide (Mimotopes, NANPNANPNANPNANPNANPNANPC) or 1μg/mL Cterm peptide (ProImmune, EPSDKHIKEYLNKIQNSLSTEWSPCSVTCGNGIQVRIKPGSANKPKDELDYANDIEKKICKMEKCS) in carbonate-bicarbonate coating buffer overnight at 4 °C. Plates were washed (0.05% v/v Tween-20 in PBS) and blocked with 2% bovine serum albumin in PBS-Tween (1 hour, room temperature). Mouse serum samples were diluted in 1% bovine serum albumin in PBS-Tween-20 and added to plate in duplicates at the appropriate dilution for the vaccine regimen and time (1.5 hours, room temperature). Plates were washed and Fc-specific goat anti-mouse IgG conjugated to alkaline phosphatase (AP) (1:5000, Sigma-Aldrich, #A1418) was added for 1 hour at room temperature. Finally, plates were washed and developed by adding p-nitrophenylphosphate at 1 mg/mL in diethanolamine buffer and optical density (OD) was read at 405 nm (BioTek ELx808, Agilent). Total IgG concentrations in serum samples were calculated as ELISA Units (EU) by interpolation against a standard curve of monoclonal 2A10 (MR4, MRA-183A, Absolute Antibody, #Ab03231) for NANP-specific or a serum pool of previously R21-immunized mice for Cterm-specific ELISA.

### Malaria challenge

Malaria challenge was carried out using transgenic *Plasmodium berghei* (*P. berghei*) parasites carrying an additional copy of the *P. falciparum* CSP gene at the 230p locus under the control of the *P. berghei* UIS4 promoter(56), produced as described previously (35, 53). For all experiments 1,000 transgenic *P. berghei* sporozoites were injected intravenously (i.v.) in a total volume of 100 µL into the lateral tail vein. From day 5 post challenge, mice were monitored for infection by thin-film blood smear (fixed in methanol and stained in 10% Giemsa for 30 min) and euthanized when >1% parasitemia was observed. If no parasites were detected on day 12 after challenge, mice were considered sterilely protected.

### Statistical Analysis

Individual-level data are presented in data file S1. All statistical analysis was performed in R (version 4.2.1). Null-hypothesis significance testing was conducted at an alpha level of 0.05. For antibody titers, non-inferiority (NI) was declared if the 95% confidence interval (CI) for a fold change was entirely above the non-inferiority threshold, which was set at 0.5 in accordance with previous reports (57, 58). Except for fig. S12, generalized additive model (GAM) fits were only used as a guide to the eye.

For analysis of in vitro kinetic profiles, time to 50% release from microcapsules in vitro was estimated as the mean of time to 50% release (obtained for each replicate by linear interpolation) and reported with 95% Student confidence intervals. For analysis of in vivo immune kinetic profiles, following previous reports (59) and recommendations (60), longitudinal antibody titers in fig. S12 were fitted after log transformation with a GAM using the mgcv package (version 1.8-40). Penalized cubic regression splines were used to model the time covariate for each regimen, with 10 knots placed evenly throughout its range. Regimen was modelled as a fixed effect, and random effects for subject and subject by time interaction were also included to account for within-subject correlations.

Marginal sampling distributions of the lag time, or the time to reach 50% peak antibody titer of the Prime regimen, were generated for each regimen, following (61), by repeatedly: (1) sampling model coefficients from the multivariate normal posterior distribution, (2) predicting log titers at 0.01 week steps, and (3) estimating lag time as the time to reach 50% of Prime peak titer. This process was repeated 10,000 times per regimen. Geometric mean titer fold change and mean additional delay, and their 95% CIs, were then estimated from the corresponding sampling distributions.

The IVIS signal was calculated as the total radiance efficiency after subtraction of residual autofluorescence. Longitudinal signals were fitted, for each mouse, to a 3-parameter logistic function by nonlinear least squares. The data shown in fig. S10 are normalized to the predicted total radiance efficiency at the first timepoint. 95% simultaneous CIs were estimated by propagating the standard error of the regression coefficients with the delta method, using a critical value obtained with the sup-t method.

For analysis of antibody titers at the times of challenge, geometric mean fold changes (Fig. 5, D and E and fig. S13, D and E) were calculated from the measured titers by bootstrapping differences of mean log-transformed titers and back-transforming the mean of the bootstrap samples. Bias-corrected and accelerated (BCa) bootstrap confidence intervals are reported (10,000 resamples). p values were calculated by inversion of the confidence interval.

Regimen efficacy, defined as sterile protection in a malaria challenge, which corresponds, in this malaria model, to the standard definition of 1 minus risk ratio relative to unvaccinated (all unvaccinated mice develop parasitemia), was modelled by a log-binomial regression, using challenge week, regimen, adjuvant and interaction between regimen and challenge week as covariates. CIs were estimated using the profile-likelihood method. p values were calculated with the likelihood ratio test.

## Supplementary Material

Data File

movie S1

Supplementary Materials

## Data Availability

All data associated with this study are in the paper or the supplementary materials. All data used to reproduce the statistical analysis and plots are saved as .csv files on a Figshare repository (DOI: 10.6084/m9.figshare.26370298). The R21 antigen can be made available by contacting A.M and completion of an MTA. VFI adjuvants can be made available through the VFI website (https://www.vaccineformulationinstitute.org/). The SMNP adjuvant can be made available through contacting the lab of Darrell Irvine (djirvine@mit.edu) and completion of an MTA.

## One sentence summary

Engineered injectable microcapsules loaded with the R21 malaria vaccine enable delayed release, potentially removing the need for booster injections.

## References

[1] Vaccination and Immunization Statistics - UNICEF DATA.

[2] WHO Immunization Data portal.

[3] (2023). Analytical Fact Sheet What are the leading causes of death in the African Region?.

[4] Bloom BR (1989). Vaccines for the Third World. Nature.

[5] O’Hagan DT, Singh M, Gupta RK (1998). Poly(lactide-co-glycolide) microparticles for the development of single-dose controlled-release vaccines. Adv Drug Deliv Rev.

[6] Johansen P, Gander B, Merkle HP, Sesardic D (2000). Ambiguities in the preclinical quality assessment of microparticulate vaccines. Trends Biotechnol.

[7] Walters AA, Krastev C, Hill AVS, Milicic A (2015). Next generation vaccines: single-dose encapsulated vaccines for improved global immunisation coverage and efficacy. Journal of Pharmacy and Pharmacology.

[8] Tzeng SY, Guarecuco R, McHugh KJ, Rose S, Rosenberg EM, Zeng Y, Langer R, Jaklenec A (2016). Thermostabilization of inactivated polio vaccine in PLGA-based microspheres for pulsatile release. Journal of Controlled Release.

[9] Yang YY, Chia HH, Chung TS (2000). Effect of preparation temperature on the characteristics and release profiles of PLGA microspheres containing protein fabricated by double-emulsion solvent extraction/evaporation method. Journal of Controlled Release.

[10] Ye M, Kim S, Park K (2010). Issues in long-term protein delivery using biodegradable microparticles. Journal of Controlled Release.

[11] Michaelides K, Prasanna M, Badhan R, Mohammed AUR, Walters A, Howard MK, Dulal P, Al-Khattawi A (2023). Single administration vaccines: delivery challenges, in vivo performance, and translational considerations. Expert Rev Vaccines.

[12] Joshi VB, Geary SM, Salem AK (2012). Biodegradable Particles as Vaccine Delivery Systems: Size Matters. AAPS J.

[13] Kanchan V, Panda AK (2007). Interactions of antigen-loaded polylactide particles with macrophages and their correlation with the immune response. Biomaterials.

[14] Amssoms K, Born PA, Beugeling M, De Clerck B, Van Gulck E, Hinrichs WLJ, Frijlink HW, Grasmeijer N, Kraus G, Sutmuller R, Simmen K (2018). Ovalbumin-containing core-shell implants suitable to obtain a delayed IgG1 antibody response in support of a biphasic pulsatile release profile in mice. PLoS One.

[15] Richards Grayson AC, Choi IS, Tyler BM, Wang PP, Brem H, Cima MJ, Langer R (2003). Multi-pulse drug delivery from a resorbable polymeric microchip device. Nature Materials.

[16] McHugh KJ, Nguyen TD, Linehan AR, Yang D, Behrens AM, Rose S, Tochka ZL, Tzeng SY, Norman JJ, Anselmo AC, Xu X (2017). Fabrication of fillable microparticles and other complex 3D microstructures. Science (1979).

[17] Tran KTM, Gavitt TD, Farrell NJ, Curry EJ, Mara AB, Patel A, Brown L, Kilpatrick S, Piotrowska R, Mishra N, Szczepanek SM (2020). Transdermal microneedles for the programmable burst release of multiple vaccine payloads. Nature Biomedical Engineering.

[18] Tu F, Lee D (2012). Controlling the stability and size of double-emulsion-templated poly(lactic-co-glycolic) acid microcapsules. Langmuir.

[19] Wu J, Yadavali S, Lee D, Issadore DA (2021). Scaling up the throughput of microfluidic droplet-based materials synthesis: A review of recent progress and outlook. Appl Phys Rev.

[20] Yadavali S, Jeong HH, Lee D, Issadore D (2018). Silicon and glass very large scale microfluidic droplet integration for terascale generation of polymer microparticles. Nature Communications.

[21] Han T, Zhang L, Xu H, Xuan J (2017). Factory-on-chip: Modularised microfluidic reactors for continuous mass production of functional materials. Chemical Engineering Journal.

[22] van der Kooij RS, Steendam R, Zuidema J, Frijlink HW, Hinrichs WLJ (2021). Microfluidic production of polymeric core-shell microspheres for the delayed pulsatile release of bovine serum albumin as a model antigen. Pharmaceutics.

[23] Datoo MS, Natama HM, Somé A, Bellamy D, Traoré O, Rouamba T, Tahita MC, Ido NFA, Yameogo P, Valia D, Millogo A (2022). Efficacy and immunogenicity of R21/Matrix-M vaccine against clinical malaria after 2 years’ follow-up in children in Burkina Faso: a phase 1/2b randomised controlled trial. Lancet Infect Dis.

[24] Willyard C (2023). The next frontier for malaria vaccination. Nature.

[25] CDC Ncird, Vaccine Administration: Needle Gauge and Length.

[26] Beirne PV, Hennessy S, Cadogan SL, Shiely F, Fitzgerald T, Macleod F (2018). Needle size for vaccination procedures in children and adolescents. Cochrane Database of Systematic Reviews.

[27] Men Y, Audran R, Thomasin C, Eberl G, Demotz S, Merkle HP, Gander B, Corradin G (1999). MHC class I- and class II-restricted processing and presentation of microencapsulated antigens. Vaccine.

[28] Zolnik BS, Burgess DJ (2007). Effect of acidic pH on PLGA microsphere degradation and release. J Control Release.

[29] Cassidy WM, Watson B, Ioli VA, Williams K, Bird S, West DJ (2001). A Randomized Trial of Alternative Two- and Three-Dose Hepatitis B Vaccination Regimens in Adolescents: Antibody Responses, Safety, and Immunologic Memory. Pediatrics.

[30] Griffin JFT, MacKintosh CG, Rodgers CR (2006). Factors influencing the protective efficacy of a BCG homologous prime-boost vaccination regime against tuberculosis. Vaccine.

[31] Park TG (1994). Degradation of poly(d,l-lactic acid) microspheres: effect of molecular weight. Journal of Controlled Release.

[32] Xu Y, Kim CS, Saylor DM, Koo D (2017). Polymer degradation and drug delivery in PLGA-based drug–polymer applications: A review of experiments and theories. J Biomed Mater Res B Appl Biomater.

[33] Walker J, Albert J, Liang D, Sun J, Schutzman R, Kumar R, White C, Beck-Broichsitter M, Schwendeman SP (2023). In vitro degradation and erosion behavior of commercial PLGAs used for controlled drug delivery. Drug Deliv Transl Res.

[34] Street D, Bangsbo J, Juel C (2001). Interstitial pH in human skeletal muscle during and after dynamic graded exercise. J Physiol.

[35] Collins KA, Snaith R, Cottingham MG, Gilbert SC, Hill AVS (2017). Enhancing protective immunity to malaria with a highly immunogenic virus-like particle vaccine. Scientific Reports.

[36] Datoo MS, Dicko A, Tinto H, Ouédraogo JB, Hamaluba M, Olotu A, Beaumont E, Ramos Lopez F, Natama HM, Weston S, Chemba M (2024). Safety and efficacy of malaria vaccine candidate R21/Matrix-M in African children: a multicentre, double-blind, randomised, phase 3 trial. The Lancet.

[37] Champion JA, Mitragotri S (2006). From the Cover: Role of target geometry in phagocytosis. Proc Natl Acad Sci U S A.

[38] Ma S, Sherwood JM, Huck WTS, Balabani S (2015). The microenvironment of double emulsions in rectangular microchannels. Lab Chip.

[39] Ma S, Huck WTS, Balabani S (2015). Deformation of double emulsions under conditions of flow cytometry hydrodynamic focusing. Lab Chip.

[40] Ma S, Sherwood JM, Huck WTS, Balabani S (2014). On the flow topology inside droplets moving in rectangular microchannels. Lab Chip.

[41] Sarmadi M, Ta C, VanLonkhuyzen AM, De Fiesta DC, Kanelli M, Sadeghi I, Behrens AM, Ingalls B, Menon N, Daristotle JL, Yu J (2022). Experimental and computational understanding of pulsatile release mechanism from biodegradable core-shell microparticles. Sci Adv.

[42] Berkland C, Pollauf E, Raman C, Silverman R, Kim K, Pack DW (2007). Macromolecule Release from Monodisperse PLG Microspheres: Control of Release Rates and Investigation of Release Mechanism. J Pharm Sci.

[43] Siepmann J, Elkharraz K, Siepmann F, Klose D (2005). How autocatalysis accelerates drug release from PLGA-based microparticles: A quantitative treatment. Biomacromolecules.

[44] Fu K, Pack DW, Klibanov AM, Langer R (2000). Visual Evidence of Acidic Environment Within Degrading Poly(lactic-co-glycolic acid) (PLGA) Microspheres. Pharmaceutical Research.

[45] McNamara HA, Idris AH, Sutton HJ, Vistein R, Flynn BJ, Cai Y, Wiehe K, Lyke KE, Chatterjee D, Kc N, Chakravarty S (2020). Antibody Feedback Limits the Expansion of B Cell Responses to Malaria Vaccination but Drives Diversification of the Humoral Response. Cell Host Microbe.

[46] Kester KE, McKinney DA, Tornieporth N, Ockenhouse CF, Heppner DG, Hall T, Krzych U, Delchambre M, Voss G, Dowler MG, Palensky J (2001). Efficacy of Recombinant Circumsporozoite Protein Vaccine Regimens against Experimental Plasmodium falciparum Malaria. J Infect Dis.

[47] Tam HH, Melo MB, Kang M, Pelet JM, Ruda VM, Foley MH, Hu JK, Kumari S, Crampton J, Baldeon AD, Sanders RW (2016). Sustained antigen availability during germinal center initiation enhances antibody responses to vaccination. Proc Natl Acad Sci U S A.

[48] Fortpied J, Collignon S, Moniotte N, Renaud F, Bayat B, Lemoine D (2020). The thermostability of the RTS,S/AS01 malaria vaccine can be increased by co-lyophilizing RTS,S and AS01. Malar J.

[49] Schmit N, Topazian HM, Natama HM, Bellamy D, Traoré O, Somé MA, Rouamba T, Tahita MC, Bonkodit M dit A, Sourabié A, Sorgho H (2024). The public health impact and cost-effectiveness of the R21/Matrix-M malaria vaccine: a mathematical modelling study. Lancet Infect Dis.

[50] R21 Malaria | WHO Prequalification of Medical Products IVDs, Medicines, Vaccines and Immunization Devices, Vector Control.

[51] Romanowsky MB, Abate AR, Rotem A, Holtze C, Weitz DA (2012). High throughput production of single core double emulsions in a parallelized microfluidic device. Lab Chip.

[52] Zhu G, Mallery SR, Schwendeman SP (2000). Stabilization of proteins encapsulated in injectable poly (lactide- co-glycolide). Nature Biotechnology.

[53] Collins KA, Brod F, Snaith R, Ulaszewska M, Longley RJ, Salman AM, Gilbert SC, Spencer AJ, Franco D, Ballou WR, Hill AVS (2021). Ultra-low dose immunization and multi-component vaccination strategies enhance protection against malaria in mice. Scientific Reports.

[54] O’Donnell JS, Isaacs A, Jakob V, Lebas C, Barnes JB, Reading PC, Young PR, Watterson D, Dubois PM, Collin N, Chappell KJ (2022). Characterization and comparison of novel adjuvants for a prefusion clamped MERS vaccine. Front Immunol.

[55] Sarmadi M, Behrens AM, McHugh KJ, Contreras HTM, Tochka ZL, Lu X, Langer R, Jaklenec A (2020). Modeling, design, and machine learning-based framework for optimal injectability of microparticle-based drug formulations. Sci Adv.

[56] Salman AM, Mogollon CM, Lin JW, Van Pul FJA, Janse CJ, Khan SM (2015). Generation of transgenic rodent malaria parasites expressing human malaria parasite proteins. Methods in Molecular Biology.

[57] Yetman RJ, Shepard JS, Duke A, Stek JE, Petrecz M, Klopfer SO, Kuter BJ, Schödel FP, Lee AW (2013). Concomitant administration of hepatitis A vaccine with measles/mumps/rubella/varicella and pneumococcal vaccines in healthy 12- to 23-month-old children. Human Vaccines & Immunotherapeutics.

[58] Garland SM, Steben M, Hernandez-Avila M, Koutsky LA, Wheeler CM, Perez G, Harper DM, Leodolter S, Tang GWK, Ferris DG, Esser MT (2007). Noninferiority of antibody response to human papillomavirus type 16 in subjects vaccinated with monovalent and quadrivalent L1 virus-like particle vaccines. Clinical and Vaccine Immunology.

[59] Wei J, Stoesser N, Matthews PC, Ayoubkhani D, Studley R, Bell I, Bell JI, Newton JN, Farrar J, Diamond I, Rourke E (2021). Antibody responses to SARS-CoV-2 vaccines in 45,965 adults from the general population of the United Kingdom. Nature Microbiology.

[60] Mundo AI, Tipton JR, Muldoon TJ (2022). Generalized additive models to analyze nonlinear trends in biomedical longitudinal data using R: Beyond repeated measures ANOVA and linear mixed models. Stat Med.

[61] Wood SN (2017). Generalized additive models: An introduction with R.

[62] Mcdonald JC, Duffy DC, Anderson JR, Chiu DT, Wu H, Schueller OJA, Whitesides GM (2000). Fabrication of microfluidic systems in poly(dimethylsiloxane). Electrophoresis.

[63] Trantidou T, Elani Y, Parsons E, Ces O (2017). Hydrophilic surface modification of PDMS for droplet microfluidics using a simple, quick, and robust method via PVA deposition. Microsystems & Nanoengineering.

[64] Panadare DC, Rathod VK (2017). Extraction of peroxidase from bitter gourd (Momordica charantia) by three phase partitioning with dimethyl carbonate (DMC) as organic phase. Process Biochemistry.

[65] Basu AS (2013). Droplet morphometry and velocimetry (DMV): a video processing software for time-resolved, label-free tracking of droplet parameters. Lab Chip.

