# Supplementary material for: Core-shell microcapsules compatible with routine injection enable prime-boost immunization against malaria with a single shot
Source: Sci Transl Med. Author manuscript; Available in PMC 2025 Aug 27. (PMC7618038; doi:10.1126/scitranslmed.adw2256)
Supplement: Supplementary Materials [file EMS206665-supplement-Supplementary_Materials.pdf]

Supplemental Materials and Methods

Microfluidic chip manufacturing

Microfluidic chips were produced by a soft lithography and polydimethylsiloxane (PDMS) replication method (62). Following computer-aided design of the microfluidic channels (AutoCAD, AutoDesk), the corresponding SU-8 soft lithography positive molds were manufactured externally by Micrux Technologies. The molds were made hydrophobic by vapor deposition of trimethylchlorosilane (TMCS, Sigma-Aldrich) to prevent sticking during PDMS replication. PDMS was mixed at a 10:1 ratio of pre-polymer to curing agent (SYLGARD 184, Dow) and poured onto the positive SU-8 mold. After de-gassing in a vacuum chamber until no air bubbles were visible, the PDMS was cured in an oven overnight at 65 °C. The PMDS layer was peeled off the mold and each chip design cut out with a razor blade. Inlets and outlets were punched with a blunt 18G needle, the chips cleaned with isopropanol, dried with nitrogen, and bonded to a flat PDMS slab by plasma exposition in a plasma chamber (HPT-200, Henniker plasma) for 3 min at 100% power. After plasma exposition, the PDMS chip design and PDMS slab were pressed together for 30 seconds and their bonding strengthened on a hot plate at 115 °C for 30 seconds.

Immediately following plasma bonding, spatially constrained hydrophilic coating was performed to ensure the hydrophilicity of the second intersection of the flow focusing design, adapted from previously reported method (63). In brief, a coating solution of 1% (w/v) polyvinyl alcohol (PVA, 30-70 kDa Mw, 87-90% hydrolyzed, Sigma-Aldrich, #P8136) in deionized water was injected from the outer inlet at 100 mbar while continuously flushing air was injected through the inner and middle inlet at 50 mbar to protect the first intersection from the coating solution. The treatment was performed for 5 minutes before flushing all channels with air and drying the chip for 5 minutes at 115 °C on a hot plate. The process was repeated 3 times to ensure durable and reliable hydrophilic coating in the relevant segment of the chip. Following coating, the microfluidic chips were left at 60 °C for a minimum of 48 hours to allow any uncoated parts to recover their hydrophobicity. Chips were then stored at room temperature and used for microfluidic emulsification within 3 months of manufacture.

Fluidics formulation

The following fluid compositions were used for the emulsification processes. Inner fluid: For in vitro assays, fluorescent dextran payloads were dissolved in 1X PBS. For in vivo injections, AF-R21 solution was mixed with R21 solution at a ratio 1:4 then completed with the corresponding adjuvant. The inner core fluid was kept on ice in the dark for the time of production. Middle fluid: Poly(D,L-lactic-co-glycolic acid) polymers (PLGA, Resomer, Evonik) were dissolved in dimethyl carbonate (DMC, Sigma-Aldrich, #517127) solvent, in a glass vial at room temperature, at the maximum observed compatible concentration with the microfluidic setup before clogging (table S1). The glass vials were agitated on a rolling shaker for 2 hours to ensure complete dissolution of the polymer. Solvent selection was performed based on toxicity, water miscibility, viscosity, PDMS compatibility and lack of denaturing effects (64). Outer fluid: 3%(w/v) PVA (9-10k Mw, 80% hydrolyzed, Sigma-Aldrich, #360627) was dissolved in 1X Dulbecco’s phosphate buffered saline (DPBS, Gibco, #14190144).

Microfluidic double emulsification

To perform microfluidic water-in-oil-in-water (W/O/W) emulsification, a pressure driven microfluidic setup was assembled. Compressed air was generated from a compressor (6-4 quiet running compressor, Jun-Air) and fed to a microfluidics dedicated pressure controller (OB1, Elveflow). Separated reservoirs containing the inner, middle, and outer fluids, supplied by Darwin Microfluidics or custom manufactured in Delrin, were precisely pressurized by the pressure controller and their liquid content pushed into the microfluidic chip through polytetrafluoroethylene (PTFE) tubing (1/32" outer diameter, 300 μm inner diameter, Darwin microfluidics) and ¼ 28" fittings. The emulsification process was monitored using an inverted microscope (DMi8, Leica) operated in brightfield, and a high-speed camera (Nova S16,Phantom). The injected pressures were set respectively to 200, 300, and 400 mbar initially, to prefill all channels and reduce risk of backflow. After 2 minutes the flow pressures were changed to the required pressure and monitored using the high-speed camera. Videos of the production were taken, and the core and outer droplet diameter and frequency measured using a dedicated software (DMV, developed at Wayne State University (65)) and ImageJ. The pressures were adjusted manually until the droplets reached the desired diameter and maximum generation frequency. Double emulsions were collected in a glass petri dish and transferred to a glass vial (22 mL) filled with outer fluid. Extraction of the solvent was performed over 48 hours at room temperature on a roller shaker, replacing the DPBS extraction media every 12 hours. Resulting microcapsules were then filtered using mini cell strainers (100 μm, pluriSelect) and stored in 2 mL of DPBS in Eppendorf tubes at 4 °C until use. If shelf-dried, the volume of DPBS was reduced to a minimum, and the Eppendorf tubes were left at room temperature for 48 hours. Quality control was performed by fluorescent microscopy imaging, as described in the corresponding section.

Batch emulsification

To produce particles by batch emulsification (BE), 100 μL of inner fluid was pipetted into 500 μL of middle fluid. The mix was vortexed at 3,000 rpm for 1 minute in a 2 mL LoBind Eppendorf tubes. The resulting water-in-oil emulsion was transferred by pipetting into 10 mL of the outer fluid in a 15 mL glass vial, and vortexed at 2,500 rpm for 1 minute. The resulting water-in-oil-in-water emulsion was left to rest for 15 minutes. Extraction of the solvent was performed over 48 hours at room temperature on a roller shaker, replacing the PBS extraction media every 12 hours. Particles were then filtered using mini cell strainers (100 μm, pluriSelect) and stored in 2 mL of PBS in Eppendorf tubes at 4 °C until use.

In vitro release assay

Produced microcapsules encapsulating dextran- tetramethylrhodamine isothiocyanate (TRITC) were divided equally into 3 to 4 aliquots, suspended in 0.01% (w/v) Tween-80 (Sigma-Aldrich, # P1754) in phosphate buffered saline (PBS) in 2 mL LoBind Eppendorf tubes, and incubated in a rotating incubator (Roto-Therm, Benchmark Scientific) kept at 37 °C at 20 rpm. The combination of the full rotation and the presence of Tween 80 surfactant in the release media prevented microcapsule aggregation and sedimentation. At relevant timepoints, depending on the PLGA formulation, microcapsules were temporarily sedimented by centrifugation for 30 seconds at 50 g, and 1 mL of supernatant was replaced with fresh releasing media to provide sink conditions. 200 μL of the removed media were used for quantification of the released payload. Image release monitoring and scanning electron microscopy (SEM) were also performed at

relevant timepoints. The effects of different microcapsule formulation parameters on the in vitro release were tested separately.

Release quantification

The amount of dextran-TRITC released from the microcapsules was measured by fluorescence spectroscopy (FLUOstar Omega, BMG Labtech), using a standard curve. The release was normalized to the initial total encapsulated content for each replicate by measuring the remaining unreleased dextran-TRITC at the experimental endpoint after mechanically breaking up the microcapsules with a cell homogenizer for 30 seconds at 30,000 rpm (IKA T10 Basic Ultra Turrax Homogenizer, Cole-Parmer) and centrifugation at 15,000 g for 10 minutes to remove PLGA debris (fig. S17). The amount released at each timepoint was corrected for the dilution resulting from the partial replacement of the releasing media.

Fluorescence microscopy imaging for microcapsule characterization and monitoring of in vitro release

For each image, 20 µL of microcapsule suspension were mixed with 200 µL of 0.01% (w/v) Tween 80 in PBS in Cellview slides (Greiner) and multi-tile imaging was performed with an inverted microscope (TiE2, Nikon, 10X objective) in the brightfield and TRITC fluorescent channel. The image tiles were processed with MATLAB Image Processing Toolbox to measure the core and shell fluorescence and the diameter of each microcapsule. Briefly, each image was smoothed by applying a bilateral filter or a gaussian filter for the brightfield and fluorescent channels, respectively. Background fluorescence was removed from the fluorescence channel, and contrast was enhanced in the brightfield channel. Each individual droplet or microcapsule was detected using a Hough transform algorithm and separated from each other by watershed segmentation in case of touching. Masking techniques were used to associate each microcapsule in brightfield with its core in fluorescence, and the diameter and fluorescence of the different regions of each microcapsule were measured for different timepoints, including immediate post-production quality control. Individual microcapsule fluorescence is reported as the maximum fluorescence corrected for the microcapsule diameter. For each microcapsule, the fluorescence profile was computed by averaging the fluorescence intensities of pixels at equal distance (normalized radius) from the core center. Coefficients of variation (CV) for the core and outer microcapsules diameter distributions were computed as the ratio of standard deviation to arithmetic mean and reported as a percentage. As the microcapsules are a single core-shell system, encapsulation efficiency was computed as the ratio (%) between the number of core and outer microcapsules detected. Fluorescence microscopy images of microcapsules containing dextran-TRITC as the model payload were produced by enhancing the contrast by contrast stretching, allowing 0.35% of pixel saturation, and overlaying the TRITC and brightfield channels. Due to the lower fluorescence intensity of the AF-R21, fluorescence microscopy images of microcapsules containing AF-R21 were produced by setting the fluorescence look-up table to a fixed scale of pixel intensity [100-600] and overlaying the fluorescent and brightfield channels.

SEM imaging of the in vitro release

The microcapsule morphology immediately after production or at different timepoints of in vitro degradation was imaged using a field emission gun scanning electron microscope (FEG-SEM, Zeiss Sigma 300) with an acceleration voltage of 2 kV. Before imaging, 10 µL of microcapsules

in suspension in PBS or release media was pipetted and spread onto a conductive adhesive carbon tape attached to an SEM pin stub. A thin film of Pd/Au coating was sputtered onto the sample (Q150R ES Dual Carbon/Sputter Coater, Quorum Technologies).

Injectability Assay

A target dose of 100 µg of encapsulated dextran-TRITC was chosen to approximate the concentration of microcapsules used for in vivo experiments with R21. Microcapsules were resuspended accordingly to contain this target dose in a 50 µL volume of injection buffer and loaded in 100 µL Gas Tight glass syringes (Hamilton). The assay was performed by injecting the target dose in PBS in an Eppendorf using 25G, 27G, or 30G needles (Terumo), replicating the assay 5 times per needle gauge condition. The microcapsule shells were then broken using a cell homogenizer (IKA T10 Basic Ultra Turrax Homogenizer, Cole-Parmer) and the released dose delivered was measured by fluorescence spectroscopy (FLUOstar Omega, BMG Labtech). The injectability was computed as the ratio between the dose delivered and the target dose.

Supplementary Figures

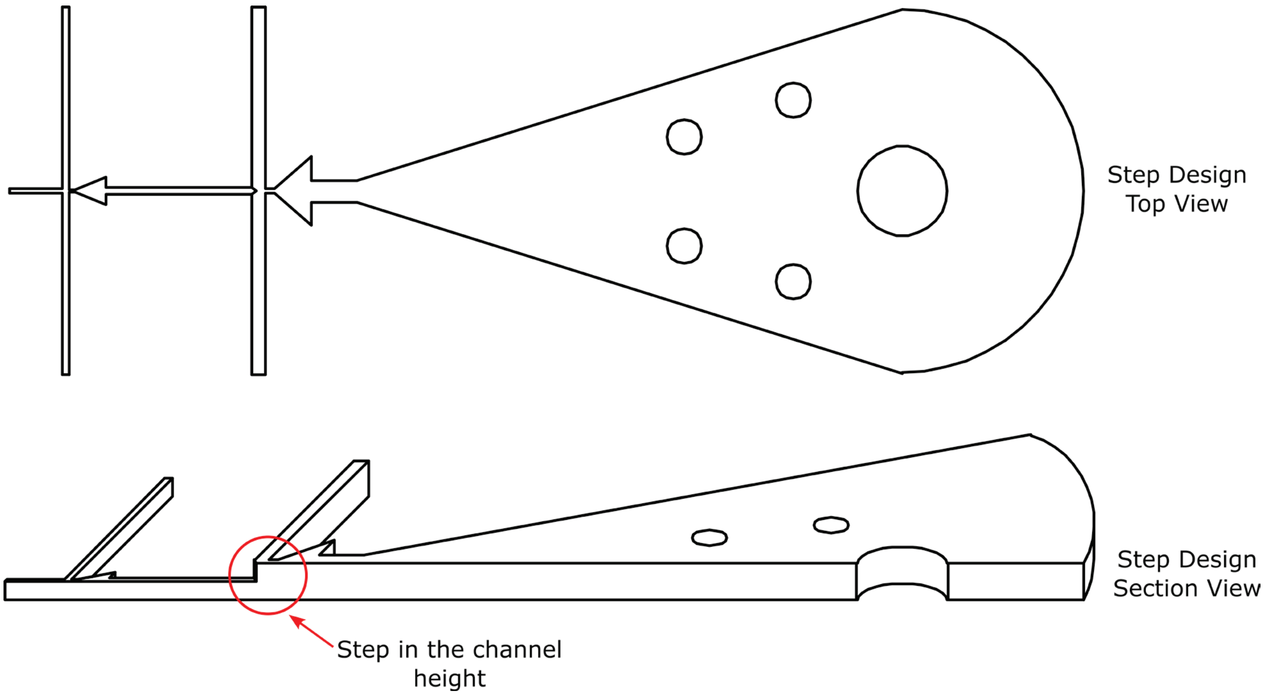

Fig. S1: Stepped microfluidic W/O/W chip design.

Top and section view of the stepped microfluidic layout developed and used in this study.

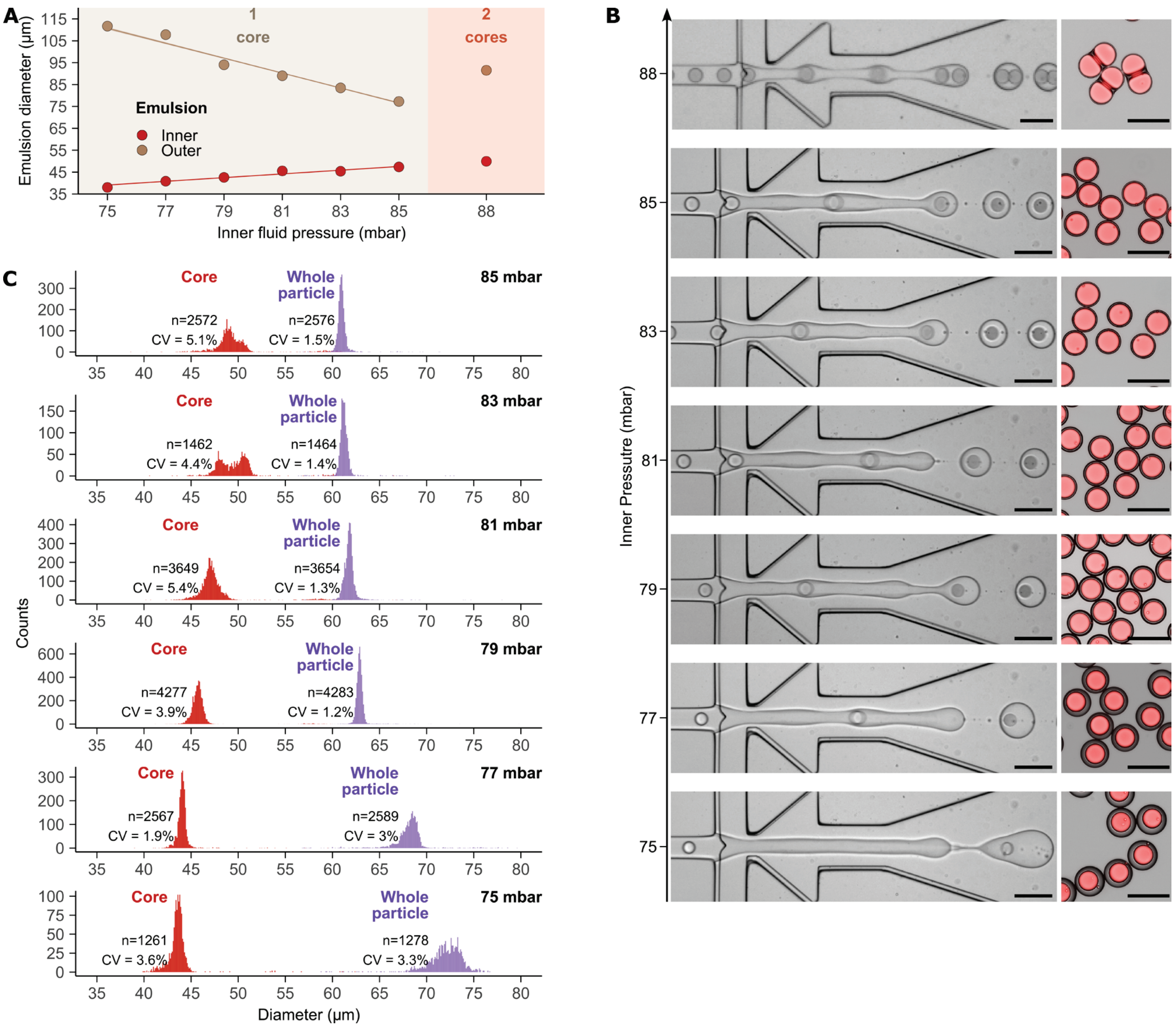

Fig. S2: The size and loading of microcapsules are controlled by the inner fluid pressure.

(A) Evolution of the core and outer diameters of the W/O/W droplets (mean ± standard deviation, n>10). **(B)** Stepped microfluidic process at different inner fluid pressures and the resulting dextran-TRITC loaded microcapsules; high-speed camera images of the emulsification process (left) and fluorescence microscopy images (right) of the corresponding microcapsules produced, TRITC signal and brightfield are overlayed. **(C)** Corresponding microcapsules (mean ± standard deviation, n and coefficient of variation, CV, displayed for each distribution) with different inner fluid pressures, using a stepped microfluidic manufacturing process. All scale bars = 100µm.

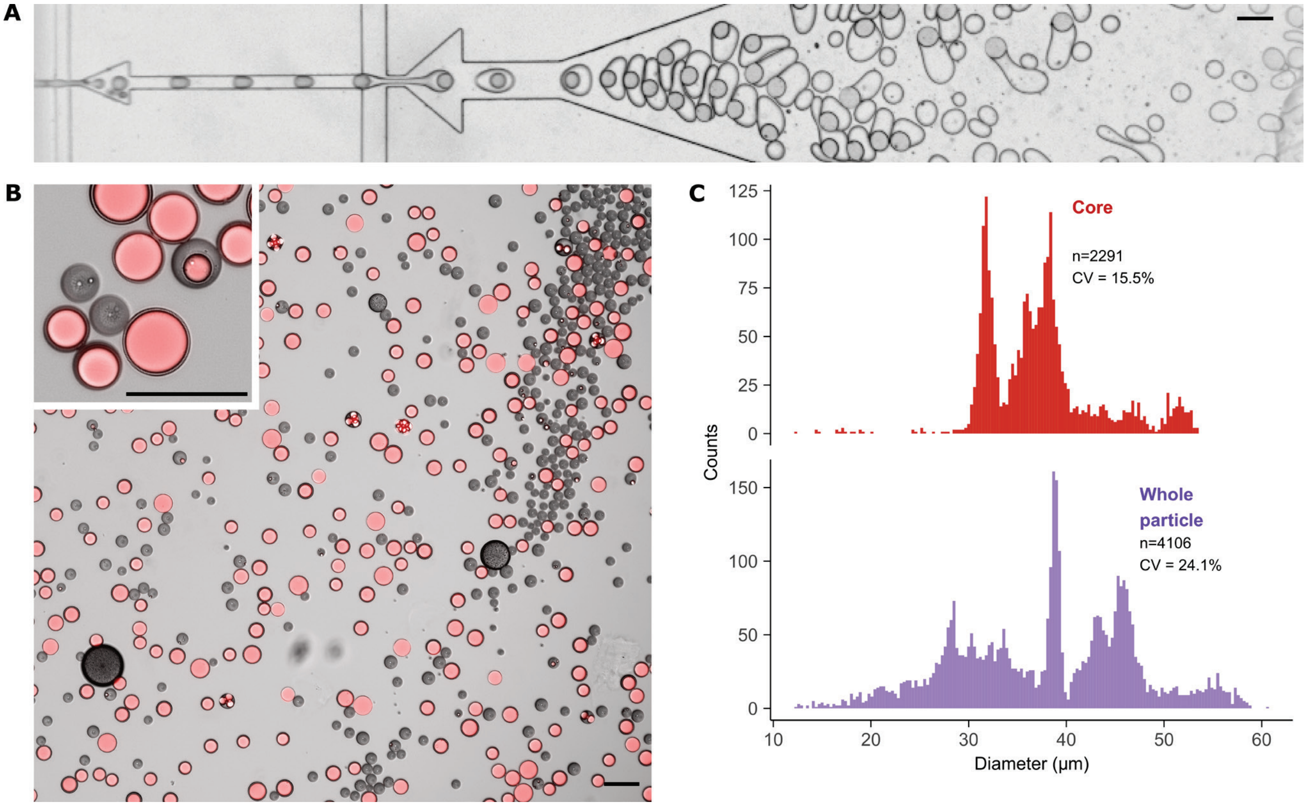

Fig. S3: A flat microfluidic design results in heterogeneous microcapsules with poor encapsulation efficiency.

(A and B) High-speed camera microscopy image of the microfluidic W/O/W emulsification process in a flat microfluidic design (A), and fluorescence microscopy images of the resulting particles following solvent extraction, using dextran-TRITC as core payload model and 7-17kDa 50:50 L:G PLGA as the shell polymer (B); TRITC and brightfield are overlayed. All scale bars = 100µm. **(C)** Corresponding core and microcapsule diameter distribution.

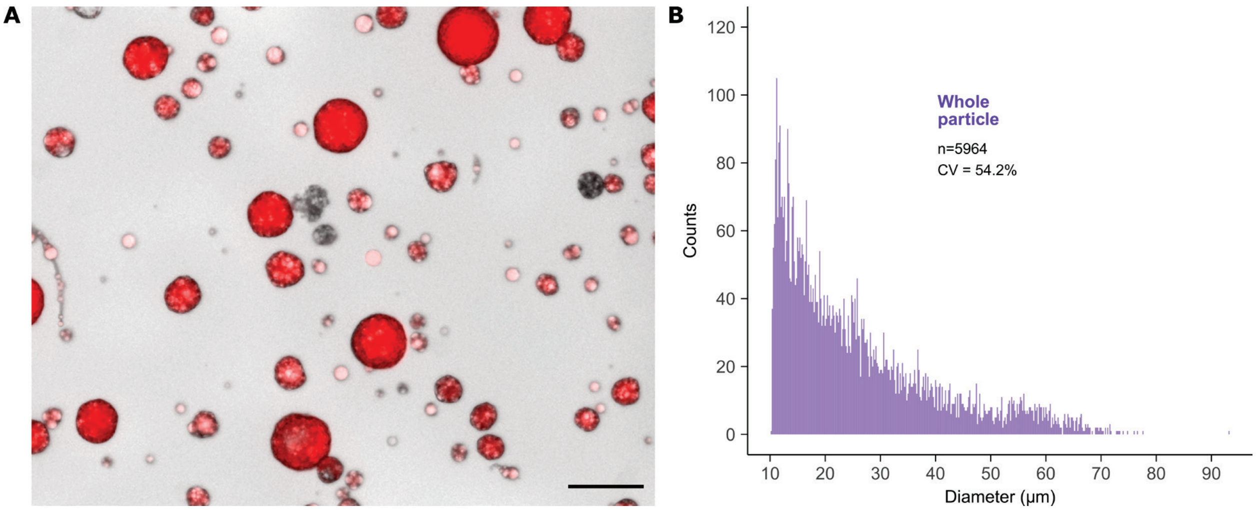

Fig. S4: Batch emulsified manufacturing results in heterogeneous particles in terms of size and encapsulation efficiency.

(A) Fluorescence microscopy image of particles produced by batch emulsification, following solvent extraction, using dextran-TRITC as core payload model and 7-17kDa 50:50 L:G PLGA as the shell polymer; TRITC and brightfield are overlayed. Scale bar = 100µm. **(B)** Corresponding core and particle diameter distribution.

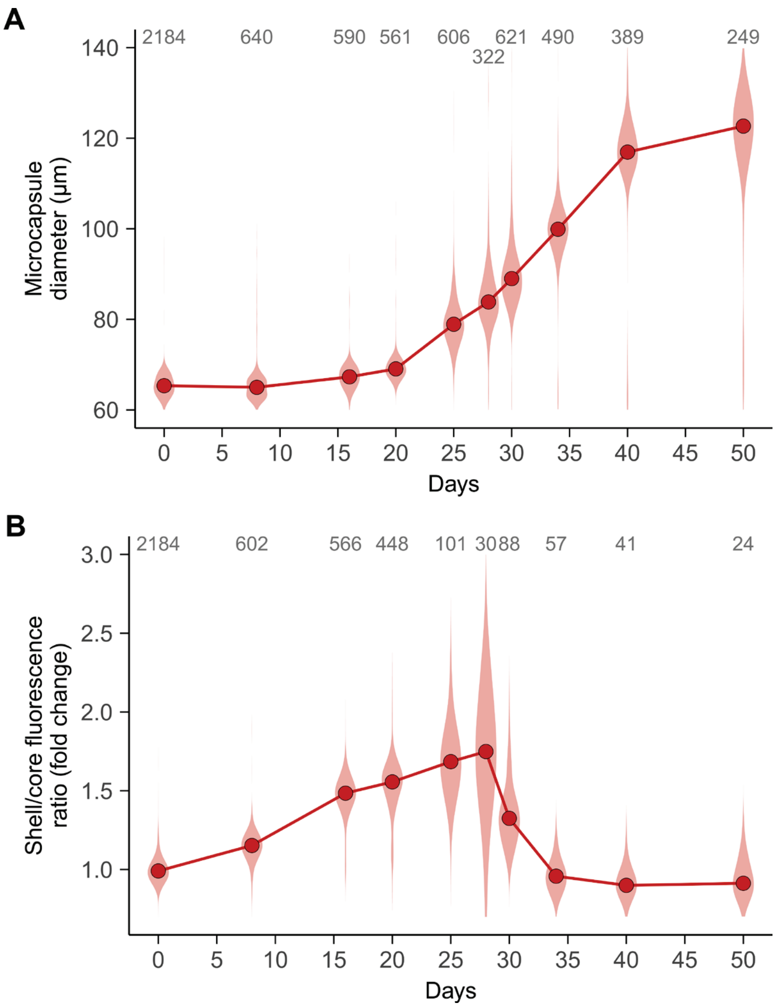

Fig. S5: Morphological changes of microcapsules with in vitro degradation.

(A and B) Evolution of the microcapsules diameter (A) and shell/core fluorescence ratio (B, loaded microcapsules only), with in vitro degradation time (median and violin plot distribution, n is shown above for each timepoint).

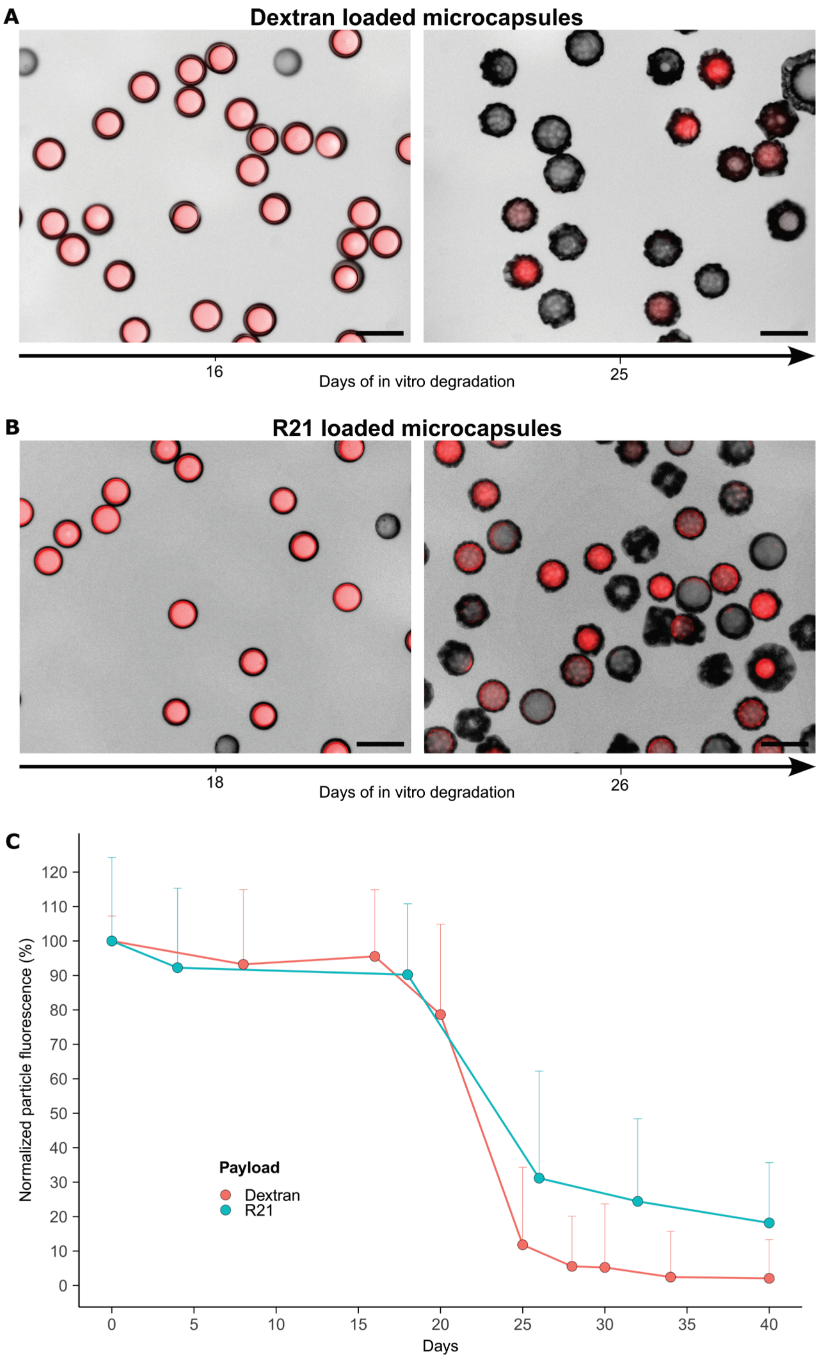

Fig. S6: R21 loaded microcapsule release kinetics are similar to dextran loaded microcapsules.

(A and B) Fluorescence microscopy of microcapsules containing dextran-TRITC (A) or AF-R21 (R21 vaccine labelled with Alexa Fluor 647, B) as the model payload, and PLGA 7-17 kDa 50:50 as the shell polymer, at different timepoints of in vitro incubation. TRITC and brightfield are overlayed. Scale bars are 100 µm. **(C)** Evolution of microcapsule fluorescence (median + standard deviation, n>300 for each point) during in vitro incubation for different payloads.

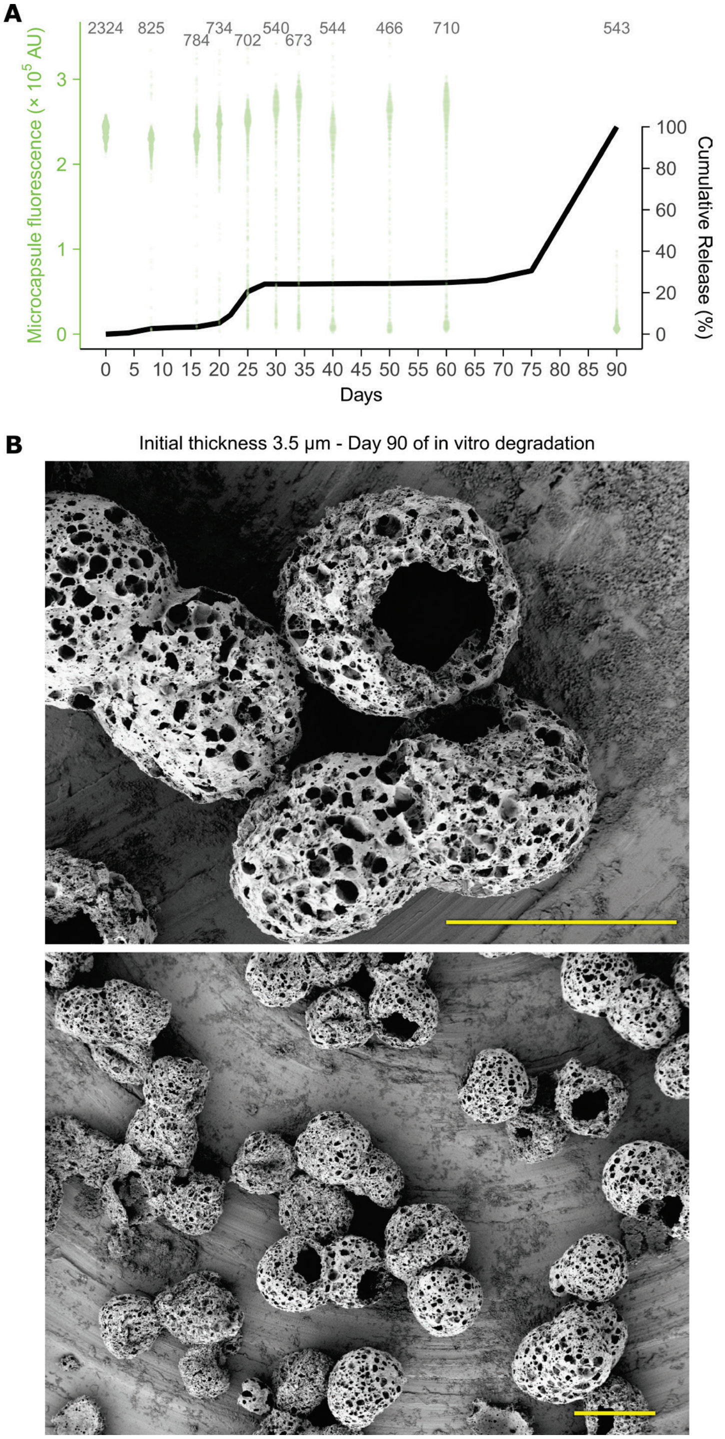

Fig. S7: Thin shell microcapsules ultimately release in vitro due to mechanical fracture.

(A) Evolution of thin shell microcapsule fluorescence during in vitro incubation (green points, n shown for each timepoint). The corresponding in vitro release curve (from Fig. 2E) is overlayed (thick black line, mean of batch replicates). AU, arbitrary units. **(B)** SEM images of microcapsules with initial shell thickness of 3.5 µm after 90 days of in vitro degradation at different magnification. Scale bar is 100 µm.

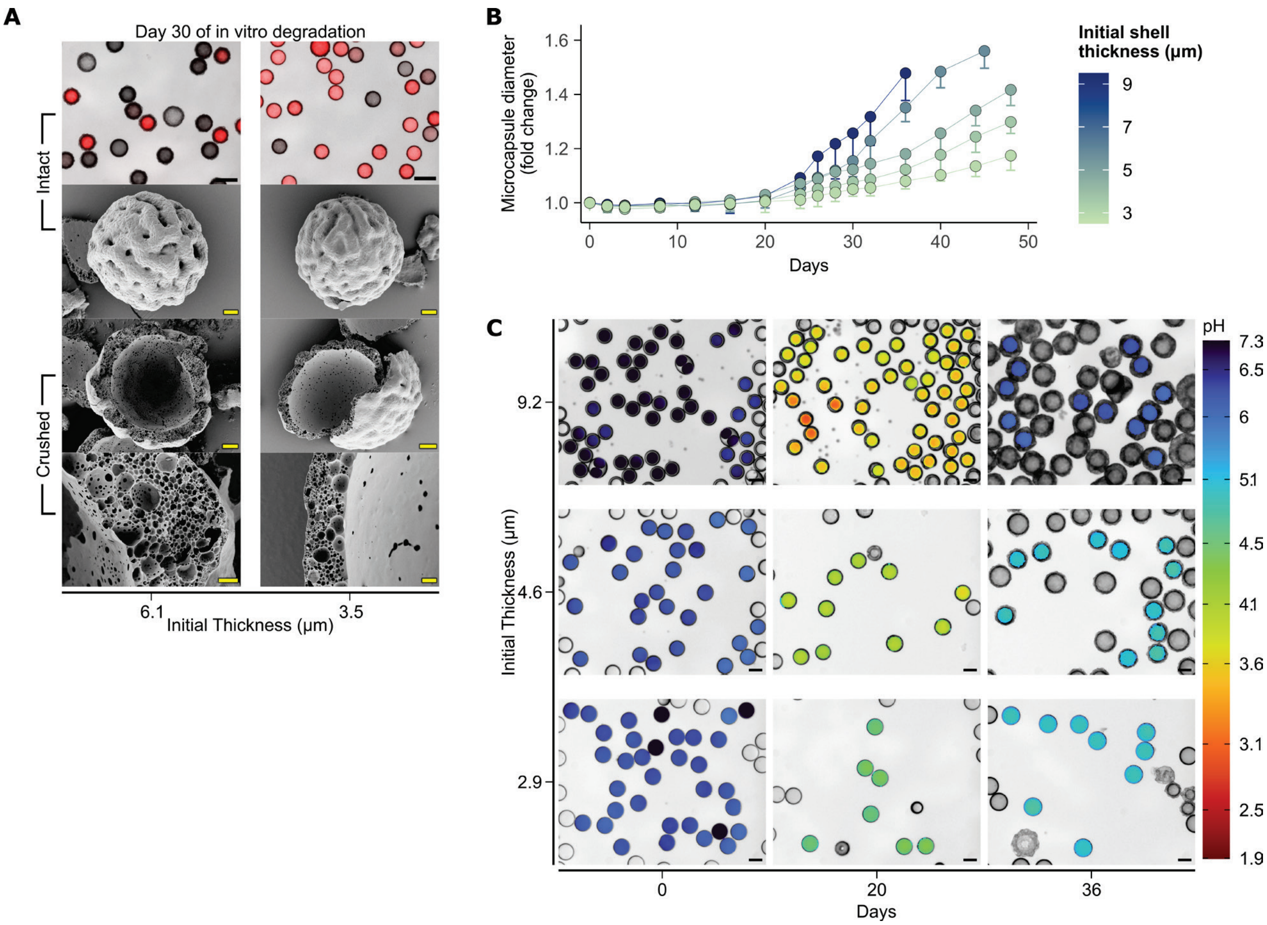

Fig. S8: Microcapsules with an initial thicker shell demonstrate a higher diameter increase and intra-shell pore network following degradation and payload release, with a greater drop in core pH.

(A) Fluorescence microscopy and SEM images of microcapsules of different initial thickness after 30 days of in vitro degradation, using dextran-TRITC/fluorescein isothiocyanate (FITC) as the core payload, and 7-17 kDa 50:50 L:G ratio PLGA as the shell polymer. TRITC signal and brightfield are overlayed. Scale bars are 100 µm (top row), 20 µm (second and third row) and 2 µm (bottom row). **(B)** Evolution of microcapsules diameter (mean + standard deviation, n>300 for each point) during in vitro incubation, for different initial shell thicknesses. **(C)** Corresponding fluorescence microscopy images of microcapsules with different initial shell thickness at different timepoints of in vitro degradation. A color map of pH calculated from the TRITC/FITC ratio is overlayed with brightfield. Scale bar is 100 µm.

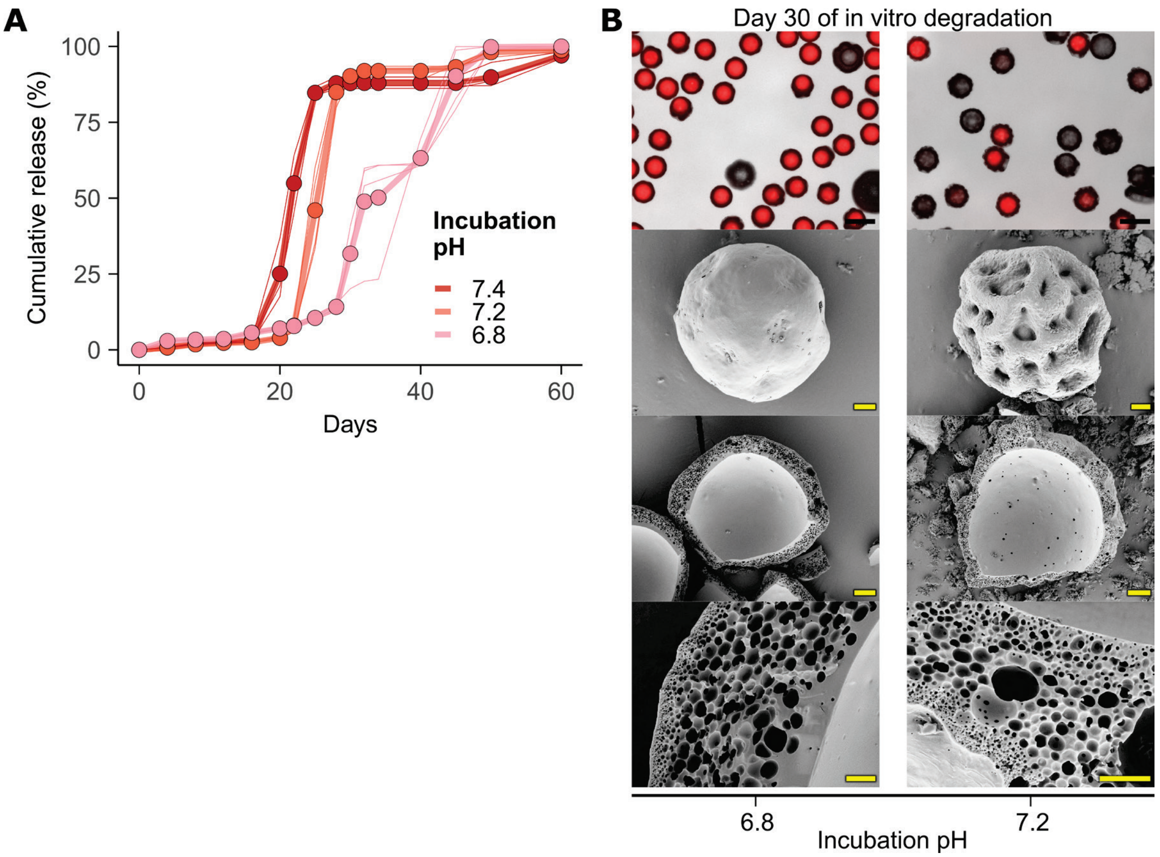

Fig. S9: Incubation pH delays the payload release from microcapsules in vitro.

(A) The effect of varying incubation pH on the kinetics of in vitro release. Mean of batch replicates (points joined by thick lines), and individual batch replicates data (thin lines) are shown (n=4). The release data for pH 7.4 are the same as displayed in Fig. 2. **(B)** Fluorescence microscopy and SEM images of microcapsules after 30 days of in vitro degradation in different incubation pH, using dextran-TRITC/FITC as the core payload, and 7-17 kDa 50:50 L:G ratio PLGA as the shell polymer; TRITC signal and brightfield are overlayed. Scale bars are 100 µm (top row), 20 µm (second and third row) and 2 µm (bottom row).

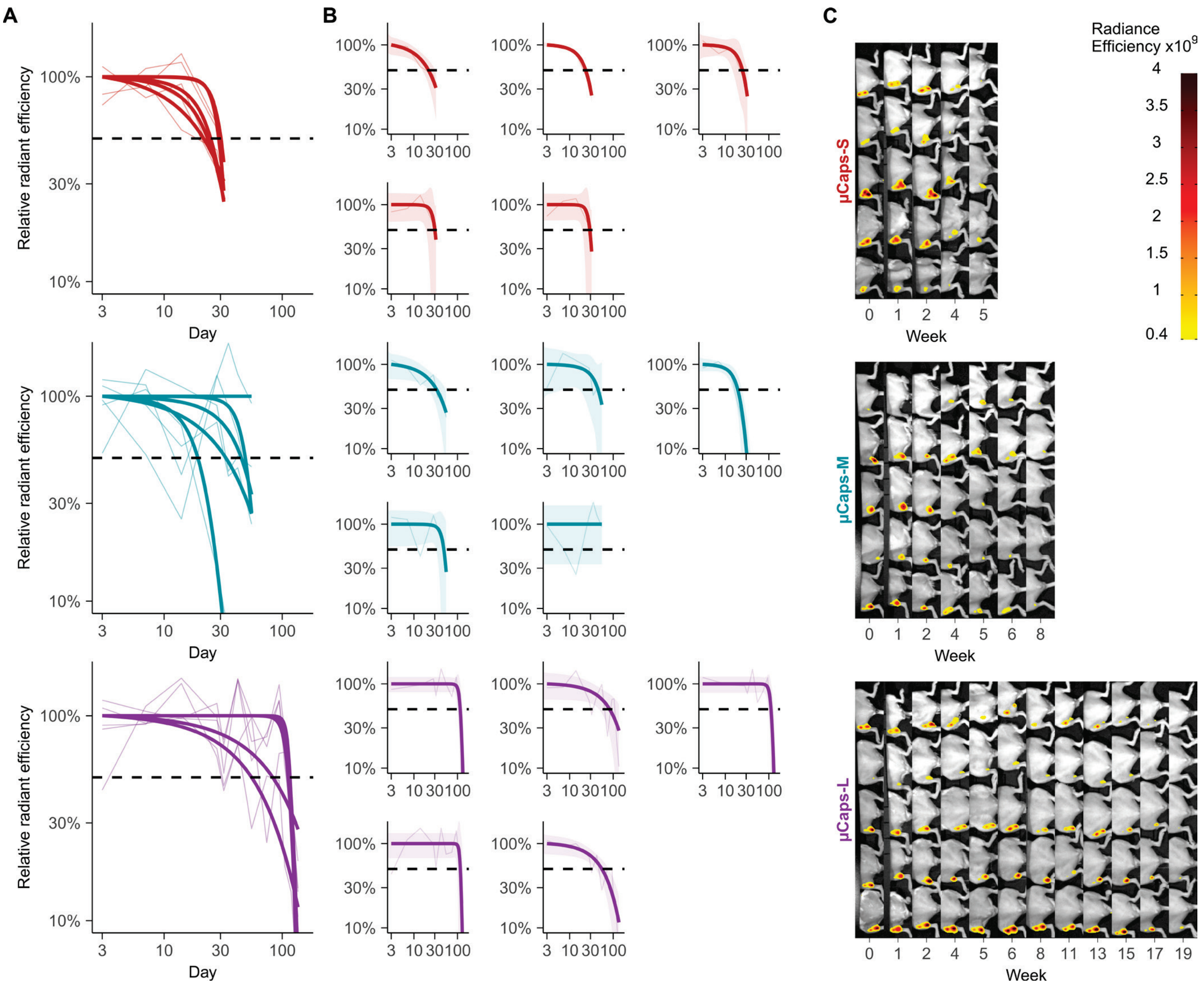

Fig. S10: Evolution of the dextran signal from different μCaps injected in mice measured by IVIS imaging.

(A to C) Fluorescent signal from 40kDa dextran-TRITC encapsulated in short delay microcapsules (μCaps-S), medium delay microcapsules (μCaps-M), and long delay microcapsules (μCaps-L), injected intramuscularly (n=5 mice per group). Individual mouse signals (thin line), corresponding fits with simultaneous 95% confidence bands (thick line and ribbons), and 50% relative radiant efficiency (dashed line) are shown. All signals are normalized to the predicted value at the first timepoint. Shown are overlayed group-level IVIS signals (A), individual mouse signals (B), and corresponding IVIS images with radiance efficiency (p/sec/cm^2/sr)/(μW/cm^2) (C).

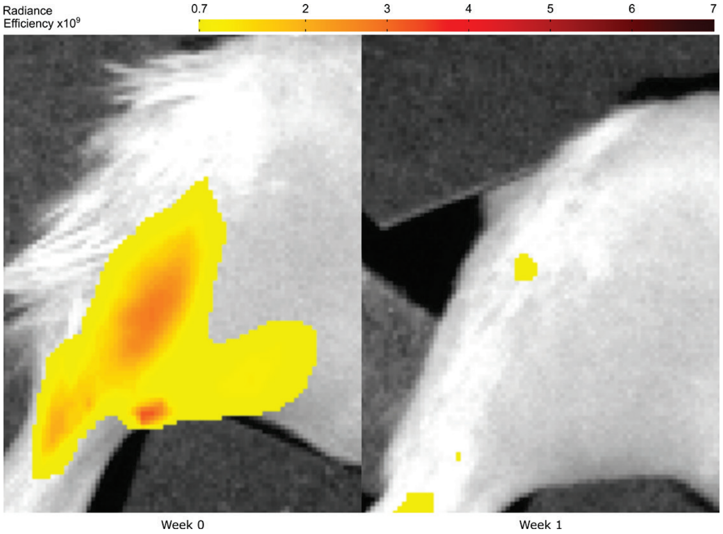

Fig. S11: Unencapsulated 40kDa dextran-TRITC is cleared within 1 week following intramuscular injection in mice.

IVIS imaging showing the radiance efficiency signal ((p/sec/cm^2/sr)/(µW/cm^2)) clearance of unencapsulated 40kDa dextran-TRITC injected intramuscularly.

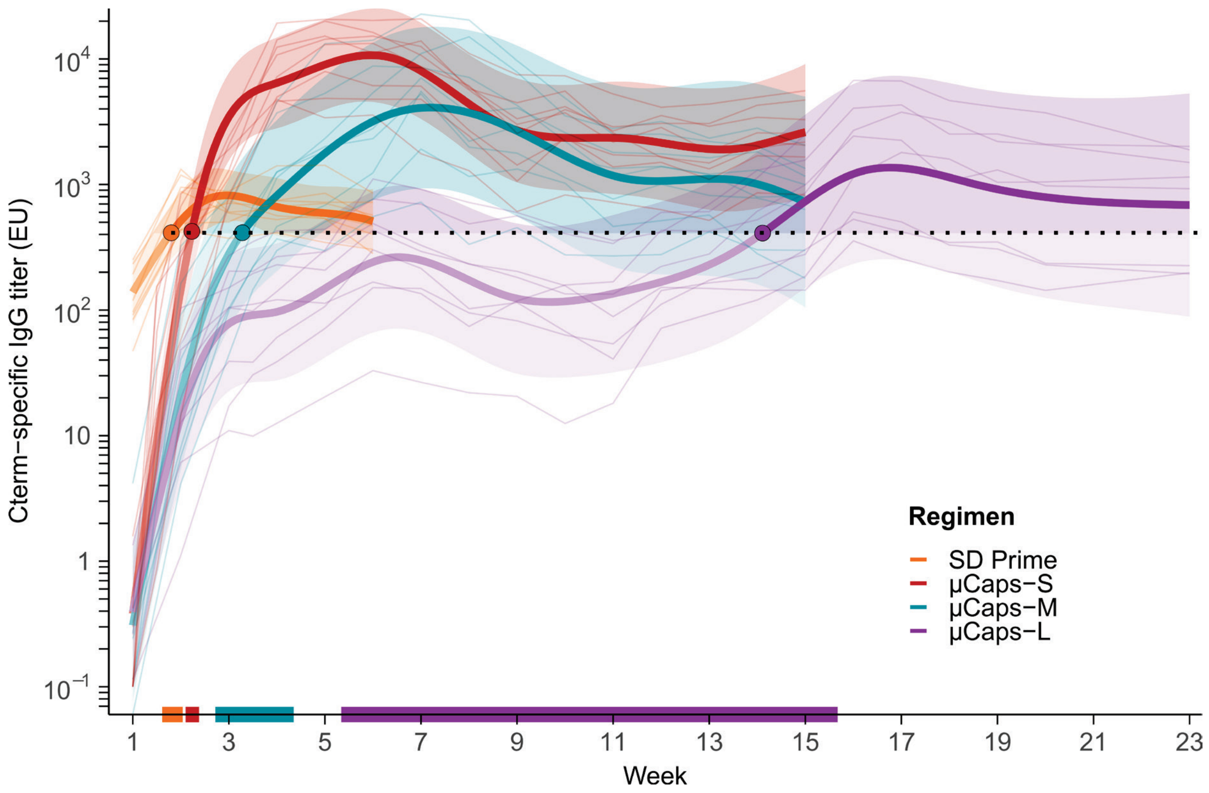

Fig. S12: C-terminus (Cterm)-specific antibody response in mice following immunization with different R21/LMQ regimen.

Cterm specific antibody titers for R21/LMQ vaccine encapsulated within µCaps-S, µCaps-M, or µCaps-L, compared with non-encapsulated single dose (SD) Prime R21/LMQ vaccine control (n=8 per group). The summary of the experimental protocol is presented in Fig. 4A. EU, enzyme-linked immunosorbent assay (ELISA) units. Regimen-level responses from generalized additive model (GAM) fits (thick lines), 95% confidence intervals (CI, ribbons) and individual responses (thin lines) are shown. The dot on each of the regimen-level curves corresponds to the time to reach 50% of the SD Prime peak titer (95% CIs are displayed as colored bands on the time axis). The dotted line represents the SD Prime peak titer value.

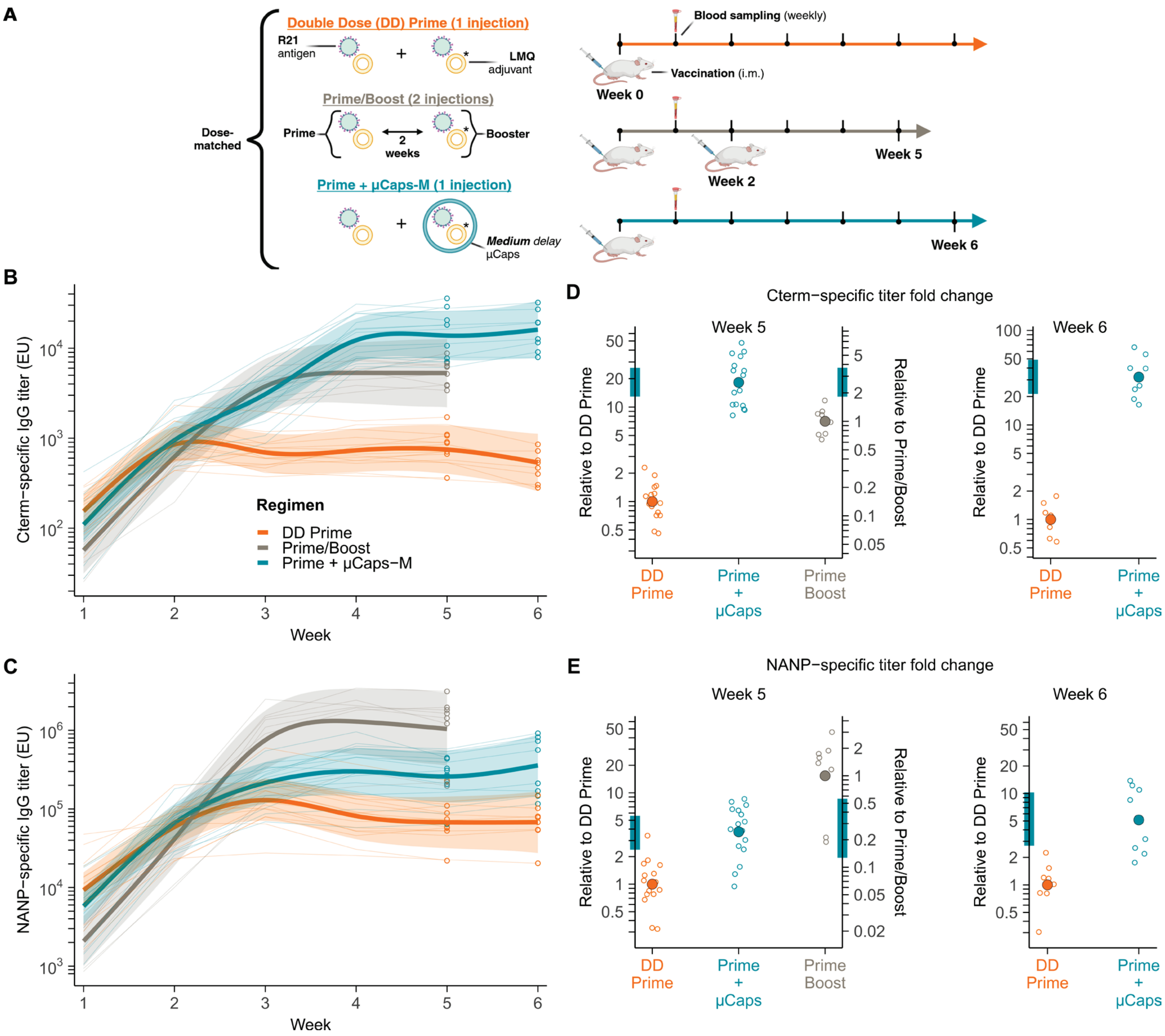

Fig. S13: Immunogenicity of the Prime+µCaps regimen using R21 in LMQ adjuvant.

(A) Summary of the experimental protocol. * indicates encapsulated LMQ adjuvant dose due to the fixed concentration of the supplied adjuvant. **(B and C)** Cterm-specific (B) and NANP-specific (C) antibody titers for different regimens of dose-matched adjuvanted R21 vaccination (Week 1 to 5: double dose (DD) Prime n=16, Prime+µCaps n=16, Prime/Boost n=8; Week 6: DD Prime n=8, Prime+µCaps n=8). The study was stopped for each mouse at the corresponding time of challenge (week 5 or week 6, Fig. 5). Regimen-level responses from GAM fits (thick lines), 95% simultaneous CI (ribbons), individual mouse responses (thin lines) and antibody titers at time of challenge (circles) are shown. **(D and E)** Fold-change in Cterm-specific (D) and NANP-specific (E) titers of Prime+µCaps compared with DD Prime or Prime/Boost at weeks 6 and 11 are plotted with individual mouse data (open circles), and their geometric mean (closed circle). Bootstrap 95% bias-corrected and accelerated (BCa) CI are displayed as colored band on the fold change axis.

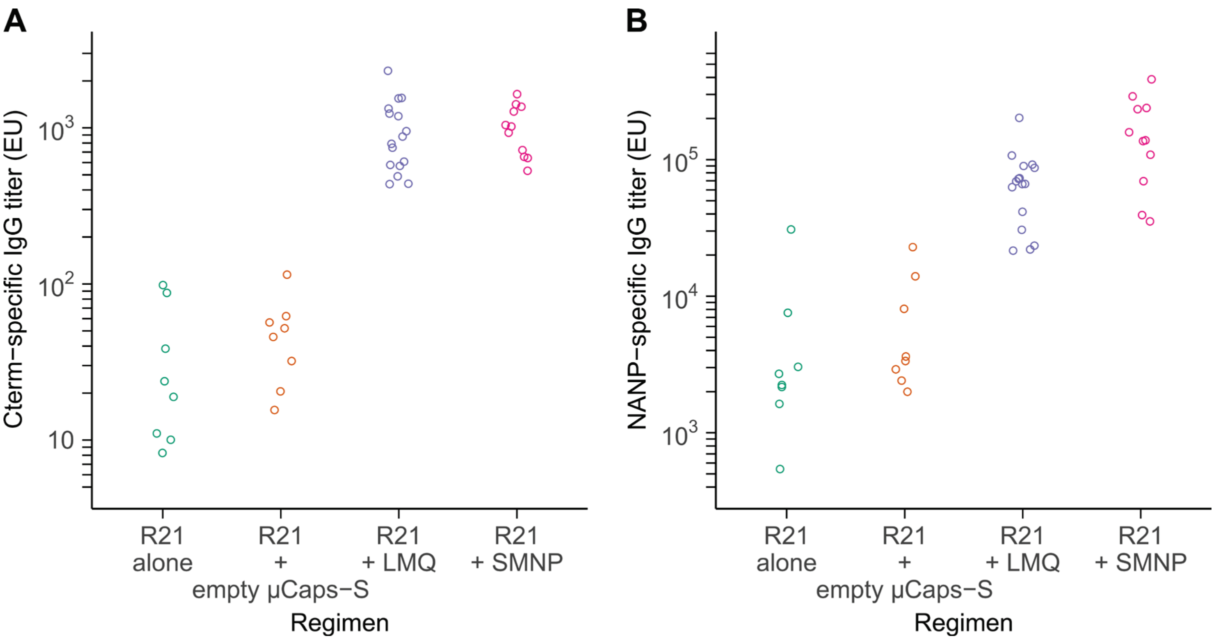

Fig. S14: Empty microcapsules do not provide an adjuvant effect.
Mice were immunized intramuscularly with R21 either alone or combined with empty µCaps-S microcapsules, and their specific antibody responses were measured at week 2 post-immunization. Data from adjuvanted R21 (from Fig. 5, B and C, fig. S13, B and C) are added as comparison. **(A and B)** Shown are Cterm-specific (A) and NANP-specific (B) antibody responses for the different regimens of R21 vaccination (R21 + LMQ n=16, R21 + SMNP n=11, other regimens n=8). Individual antibody titers (circles) are shown.

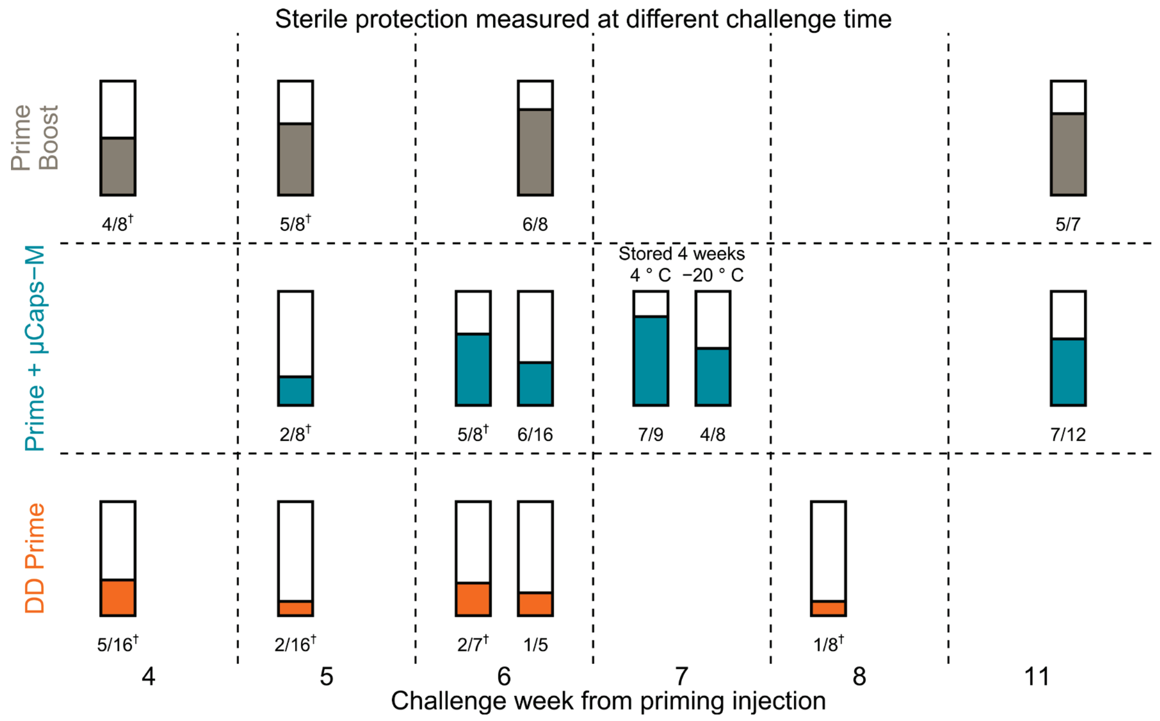

Fig. S15: Observed sterile protection depending on the regimen and time of challenge.

Sterile protection from malaria challenge performed at different intervals from priming injection, in separate groups receiving different vaccine regimens (see Fig. 6A). Each color bar corresponds to the rate of sterile protection in BALB/c mice immunized with the indicated regimen. Ratios of protected/total number of mice are displayed below each bar. R21 was adjuvanted with either SMNP or LMQ (†).

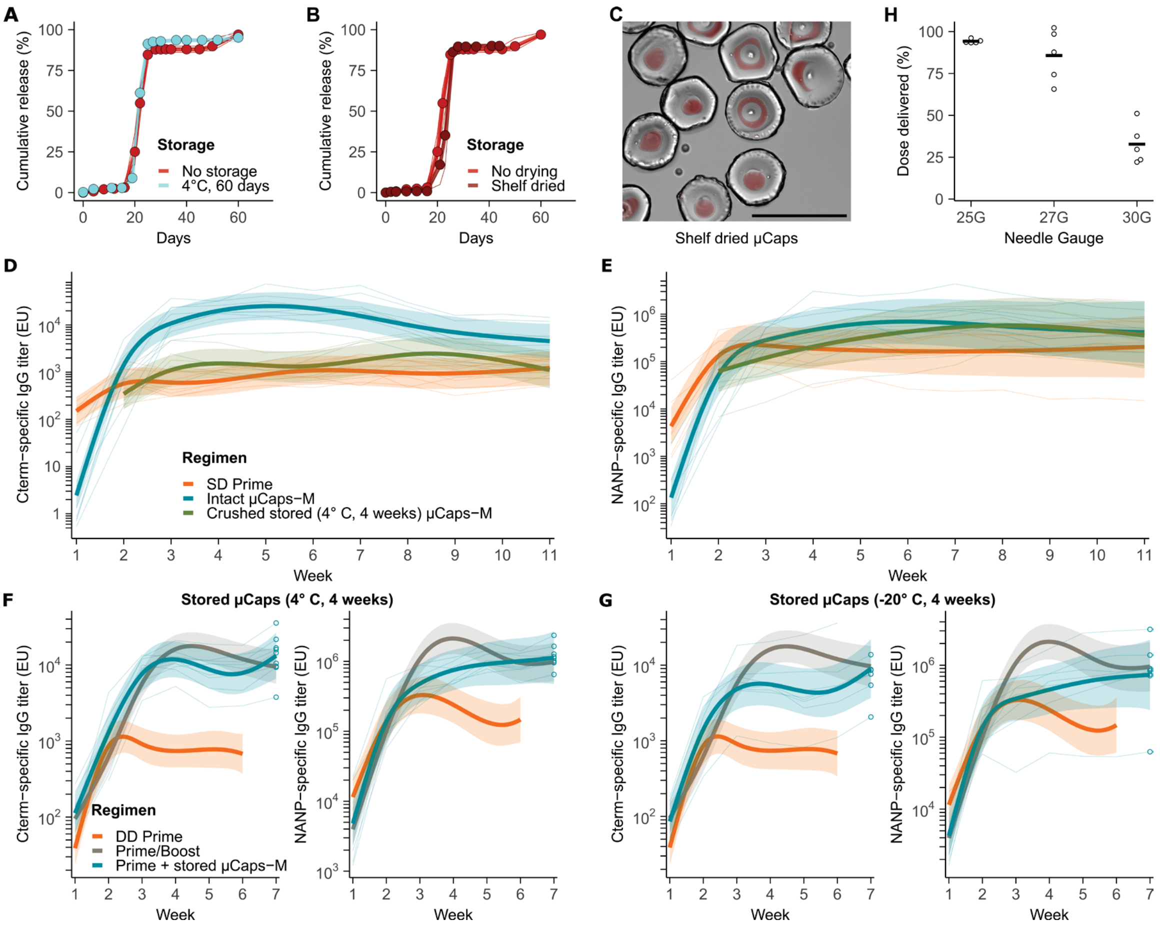

Fig. S16: Microcapsules meet technical requirements for deployment in malaria endemic countries.

(A and B) Effect of storing microcapsules for 60 days at 4°C (A), or shelf drying for 48 hours at room temperature (B) on in vitro release kinetics, using 50 mg/mL 40 kDa dextran-TRITC as the payload, compared with freshly prepared microcapsules. **(C)** Fluorescence microscopy image of shelf-dried microcapsules (7-17 kDa 50:50 L:G) immediately after reconstitution in DPBS, with dextran-TRITC as model payload; TRITC signal and brightfield are overlayed. Scale bar is 100 µm. **(D and E)** Cterm-specific (D) and NANP-specific (E) antibody responses for different regimens of dose-matched adjuvanted R21 vaccination are shown (crushed, n=7; rest, n=8). Regimen-level responses from GAM fits (thick lines), 95% simultaneous CI (ribbons), and individual mouse responses (thin lines) are shown. Intact µCaps-M were injected immediately after production. Crushed µCaps-M were kept stored for 4 weeks at 4 °C and broken by mechanical homogenization before injection. **(F and G)** Cterm-specific and NANP-specific antibody responses for dose-matched adjuvanted R21 regimens of Prime+µCaps-M after 4 weeks of storage at 4°C (F, n=9) or -20 °C (G, n=8). Regimen-level responses from GAM fits (thick lines), 95% simultaneous CI (ribbons), individual mouse responses (thin lines) and antibody titers at time of challenge (circles) are shown. Regimen-level titers of DD Prime and Prime/Boost regimen from Fig. 5 are displayed for reference. **(H)** Percentage of target dose (100 µg) of dextran-TRITC recovered after injection with different gauge needles (n=5 per group). Individual repeats (circles) and means (crossbars) are shown.

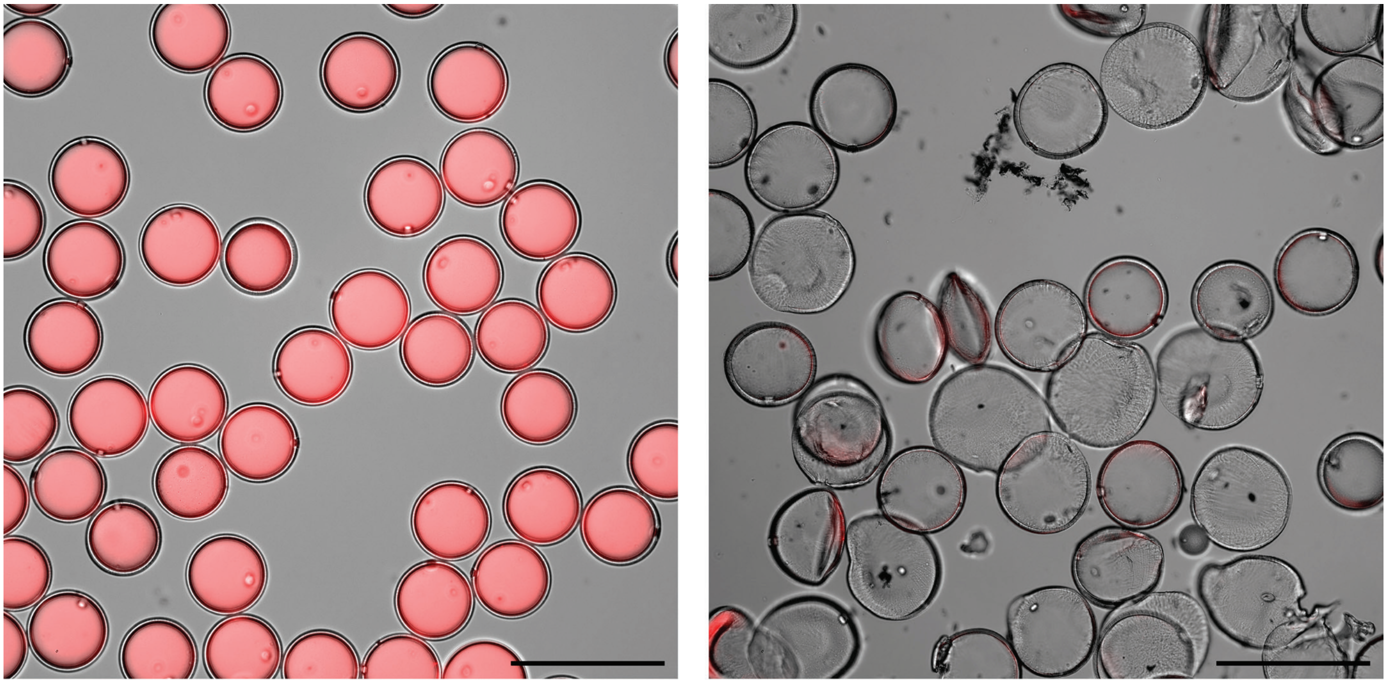

Intact microcapsules, before the mechanical break

Broken microcapsules, using a cell homogenizer to induce the mechanical break

Fig. S17: Mechanical homogenization allows the break-up and full release of the payload from microcapsules.

Fluorescence microscopy image of microcapsules loaded with a mix of AF-R21 and R21 (10X objective, AF and brightfield channels are overlayed), produced by microfluidic double emulsification, and broken up mechanically using a cell homogenizer for 30 seconds. Scale bar = 100µm.

Table S1: Summary of the PLGA concentrations used for the middle fluid formulation.

Polymer name	Mw (kDa)	End cap	Lactide:Glycolide ratio	Concentration %(w/v)
**Resomer R 502**	7-17	ester	50:50	17.75
**Resomer R 505**	54-69	ester	50:50	7.5
**Resomer R 753 S**	24-38	ester	75:25	12
**Resomer R 202 S**	7-17	ester	100:00	17.75

Movie S1: Stepped versus flat microfluidic emulsification process.

High-speed camera microscopy video of the microfluidic W/O/W emulsification process in a stepped (top) or flat (bottom) microfluidic design (slowed down 167 times). The size and loading of microcapsules are controlled by the inner fluid pressure.

Data file S1: Individual-level data.
